# Evidence of ESBL plasmid transfer and selective persistence of multiple host-associated *Escherichia coli* isolates in a chicken cecal fermentation model

**DOI:** 10.1128/aem.00822-25

**Published:** 2025-09-19

**Authors:** J. Leng, M. Ferrandis-Vila, R. Oldenkamp, J. W. Mehat, A. S. Fivian-Hughes, S. Kumar Tiwari, B. Van der Putten, V. Trung Nguyen, A. Bethe, J. Clark, P. Singh, T. Semmler, S. Schwarz, J. Alvarez, N. T. Hoa, M. Bootsma, C. Menge, C. Berens, C. Schultsz, J. M. Ritchie, R. M. La Ragione

**Affiliations:** 1 Faculty of Health and Medical Sciences, School of Veterinary Medicine, University of Surrey 417508, Guildford, United Kingdom; 2 Institute of Integrative Biology, University of Liverpool, Liverpool, United Kingdom; 3 Friedrich-Loeffler-Institut, Institute of Molecular Pathogenesis, Jena, Germany; 4 Faculty of Science, Amsterdam Institute for Life and Environment (A-LIFE), Vrije Universiteit Amsterdam 1190, Amsterdam, the Netherlands; 5 Amsterdam Institute for Global Health and Development (AIGHD), University of Amsterdam 1234, Amsterdam, the Netherlands; 6 Faculty of Health and Medical Sciences, School of Biosciences, University of Surrey 3660, Guildford, United Kingdom; 7 Genome Sequencing and Genomic Epidemiology, Robert Koch Institute 9222, Berlin, Germany; 8 Gut Microbes and Health Institute Strategic Program, Quadram Institute Bioscience, Norwich Research Park, Norwich, United Kingdom; 9 Amsterdam UMC, University of Amsterdam 1234, Amsterdam, the Netherlands; 10 Oxford University Clinical Research Unit Vietnam 160913, Ho Chi Minh City, Vietnam; 11 Institute of Microbiology and Epizootics, School of Veterinary Medicine, Freie Universität Berlin 9166, Berlin, Germany; 12 Veterinary Centre for Resistance Research (TZR), School of Veterinary Medicine, Freie Universität Berlin 9166, Berlin, Germany; 13 Department of Environmental Hygiene, German Environment Agency 39417, Berlin, Germany; 14 Epsom and St Helier University Hospitals NHS Trust, Epsom, United Kingdom; 15 Watford General Hospital 156734, Watford, United Kingdom; 16 VISAVET, Health Surveillance Centre, Complutense University, Madrid, Spain; 17 Depatment of Animal Health, Faculty of Veterinary Medicine, Complutense University of Madrid 734901, Madrid, Spain; 18 Centre for Tropical Medicine and Global Health, Nuffield Department of Medicine, University of Oxford 105596, Oxford, United Kingdom; 19 Biomedical Research Center, Pham Ngoc Thach University of Medicine 384732, Ho Chi Minh City, Vietnam; 20 Department of Mathematics, Faculty of Sciences, Utrecht University 368196, Utrecht, the Netherlands; 21 University Medical Center Utrecht, Utrecht University 8125, Utrecht, the Netherlands; Universita degli Studi di Napoli Federico II, Portici, Italy

**Keywords:** *Escherichia coli*, ESBL, gut microbiome, fermentation, antimicrobial resistance, chicken

## Abstract

The guts of animals and humans harbor diverse microbial communities that are regularly exposed to bacteria originating from food, water, and their surroundings. Species such as *Escherichia coli* are adept at colonizing multiple hosts, along with surviving in the environment. By encoding pathogenic traits and transmissible forms of antimicrobial resistance (AMR), *E. coli* can also pose a zoonotic risk. Our understanding of the factors that govern host residency is limited. Here, we used a chicken cecal fermentation model to study survival and the AMR transfer potential of 17 host-associated extended-spectrum β-lactamase (ESBL)-producing *E. coli* isolates. Vessels containing chicken cecal contents were stabilized for 4 days before the addition of a cocktail comprising ESBL-producing *E. coli* obtained from human, cattle, pig, and chicken hosts. Consecutive sampling showed that pig and cattle-associated isolates persisted in most vessels, although the recovery of all isolates declined over time. Increasing the inoculum dose or adding ceftiofur helped to stabilize populations of ESBL *E. coli* within the vessels, although this did not result in outgrowth of resistant populations in all vessels. Sequencing revealed that most new ESBL-producing *E. coli* recovered during the study acquired a *bla*_CTX-M-1_ plasmid from a single ESBL *E. coli* included in the cocktail that lacked host-specific traits (generalist). Our data highlight that isolate-specific differences in the *E. coli* genome composition likely explain the persistence of specific clones and efficiency of plasmid transfer, both of which could impact the spread of AMR in complex communities.

**IMPORTANCE:**

There are few insights into how host-associated *Escherichia coli* behave within the gut environment of other hosts. *E. coli* isolates that are immigrants to the gastrointestinal system of humans and animals have the potential to transfer their resistance to other native bacteria. A better understanding of this process is needed to assess how the gastrointestinal environment could serve as a reservoir and a melting pot of new, multidrug-resistant *E. coli* isolates.

## INTRODUCTION

The gut microbiota of animals and humans is regularly exposed to bacteria originating from food, water, the environment, or other hosts (1, 2). Some microorganisms behave as transient visitors that are rapidly lost, whereas others establish and become part of the resident microbiota (3, 4). Phenotypic traits such as motility (5), metabolic adaptability (6), and adaptation to substances such as bile (7) likely contribute to residency. Moreover, bacterial defense strategies, including the ability to produce bacteriocins, could help isolates compete within a host (8). Individual replication rates and an ability to acquire or lose genetic material may also confer advantages over less successful bacteria (9). Finally, inter- and intra-species interactions could impact population dynamics, with individual populations negatively affected by competition for resources (10) or positively affected by cross-feeding (11).

*Escherichia coli* is a well-known inhabitant of the human and animal intestine, where it may exist as a commensal or pathogenic member of the microbiota (12). There is growing interest in *E. coli* population dynamics due to its propensity for horizontal transfer of antimicrobial resistance (AMR) genes, its prevalence and pathogenesis in multiple hosts, and its survival in the natural environment. Yet the mechanisms determining whether *E. coli* from one host establishes in a new host species are not clear. Several studies suggest that certain clones of *E. coli* exhibit host preference so that they are more likely to become resident within the human intestine (4, 13). Moreover, high-resolution, isolate-level analyses of *E. coli* originating from humans and animals indicate that the two populations are genetically distinct (14–17). Consistent with this, genome-wide association studies (18) and logistic regression analysis of single nucleotide polymorphisms (19) have found evidence for host-specific determinants, although data from experimental mixed-isolate infections is generally lacking.

Resistance to third-generation cephalosporins due to plasmid-encoded extended-spectrum β-lactamases (ESBLs) is commonly detected in bacteria present in the mammalian gastrointestinal tract (20). For example, 16.5% of healthy individuals present in the community (21) and 10%–45% of pigs and cattle (22–27) were found to harbor ESBL *E. coli*. The zoonotic risks associated with ESBL *E. coli* from different hosts are not clear, and quantitative data on the potential for plasmid transfer in the gastrointestinal system are limited. Transfer of ESBL-encoding plasmids from avian-derived *E. coli* isolates to human commensal *E. coli* has previously been observed using *in vitro* models of the human gastrointestinal system (28, 29), but data from food-producing animal models are rare.

*In vitro* models using feces/gut content from humans or animals can offer a reproducible and controlled way to study the bacterial populations in the gut without the need for *in vivo* animal models. Here, we utilized a chicken cecal fermentation model to simultaneously monitor the ability of 17 previously described host-associated ESBL *E. coli* (18) to persist in the presence and absence of antimicrobial selection pressure and used whole-genome sequencing to determine which isolate(s) transferred their ESBL-encoding plasmid to the native *E. coli* present in the community. Collectively, our data indicated that the chicken-associated isolate was no better at persisting than isolates associated with cattle, pigs, or humans, and that an isolate lacking host-specific traits (18) appeared largely responsible for the spread of an ESBL plasmid to native *E. coli* within the cecal community.

## RESULTS

### Validating the use of pooled chicken cecal content as a model inoculum

**Fig 1:**
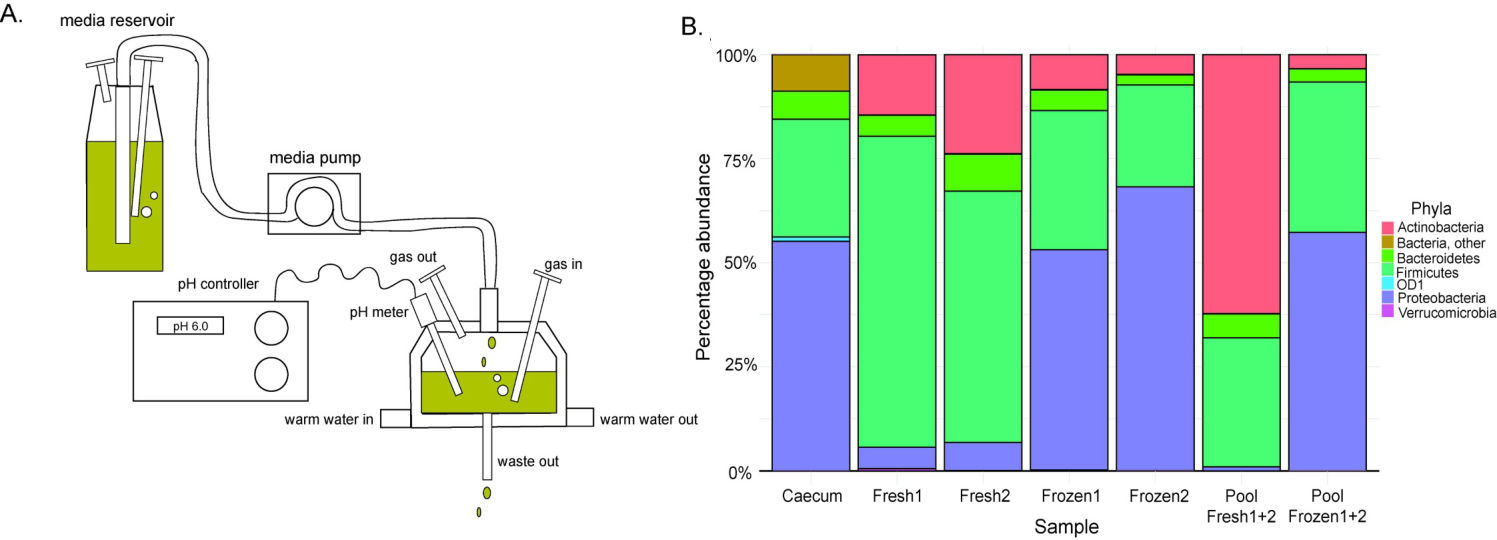
Overview and microbial content of a continuous flow chicken cecal fermentation model. (**A**) Schematic representation of the single vessel model maintained under anaerobic conditions with automatic adjustment for pH and temperature. (**B**) Bacterial composition based on 16S rRNA gene sequencing of samples from the chicken ceca and from the model after 4 days of fermentation. Bars represent the mean percentage abundance for bacterial phyla found in the chicken cecum (*N* = 3), two vessels inoculated with fresh cecal content from two independent chickens (Fresh1, Fresh2), two vessels inoculated with content Fresh1 and Fresh2 combined (Pool Fresh 1+2), vessels inoculated with cecal content from independent chickens, frozen at the time of collection and subsequently re-used (Frozen1 and Frozen2), and two vessels inoculated with a frozen pooled sample (Pool Frozen 1+2). Duplicate vessels were run for each condition.

We used a previously described single-stage, continuous flow chicken cecal fermentation model (30) in our study (Fig. 1A). To facilitate model operation, we evaluated whether frozen, pooled chicken cecal content could replace the use of freshly collected content as an inoculum. As can be seen in Fig. 1B, the bacterial composition in vessels seeded with frozen cecal samples, individual or pooled, after 4 days of incubation, more closely resembled the profile in the cecum than when fresh content was used. For example, the individual vessels inoculated with frozen material contained 52%–68% *Proteobacteria* and 24%–36% *Firmicutes*, which were comparable to the proportions identified in the ceca (55% and 28%, respectively). In contrast, the two vessels inoculated with fresh cecal samples had 60%–75% *Firmicutes*, while *Actinobacteria*, which were identified as <1% in the ceca, increased to 62% in the pooled fresh sample. The bacterial class composition of samples from frozen pooled chicken ceca was also observed to be most similar to those identified in the cecal samples (Item S1). The relative abundance of the three most common bacterial classes identified in the models inoculated with pooled frozen cecal content was 40% *Clostridia*, 23% *Gammaproteobacteria*, and 24% *Alphaproteobacteria*. This was comparable to 28% *Clostridia*, 24% *Gammaproteobacteria*, and 28% *Alphaproteobacteria* identified in the chicken cecal samples analyzed. Thus, frozen pooled chicken cecal content was used as an inoculum in all subsequent experiments.

### Immigrant *E. coli* isolates were outcompeted by the native cecal community

**Fig 2:**
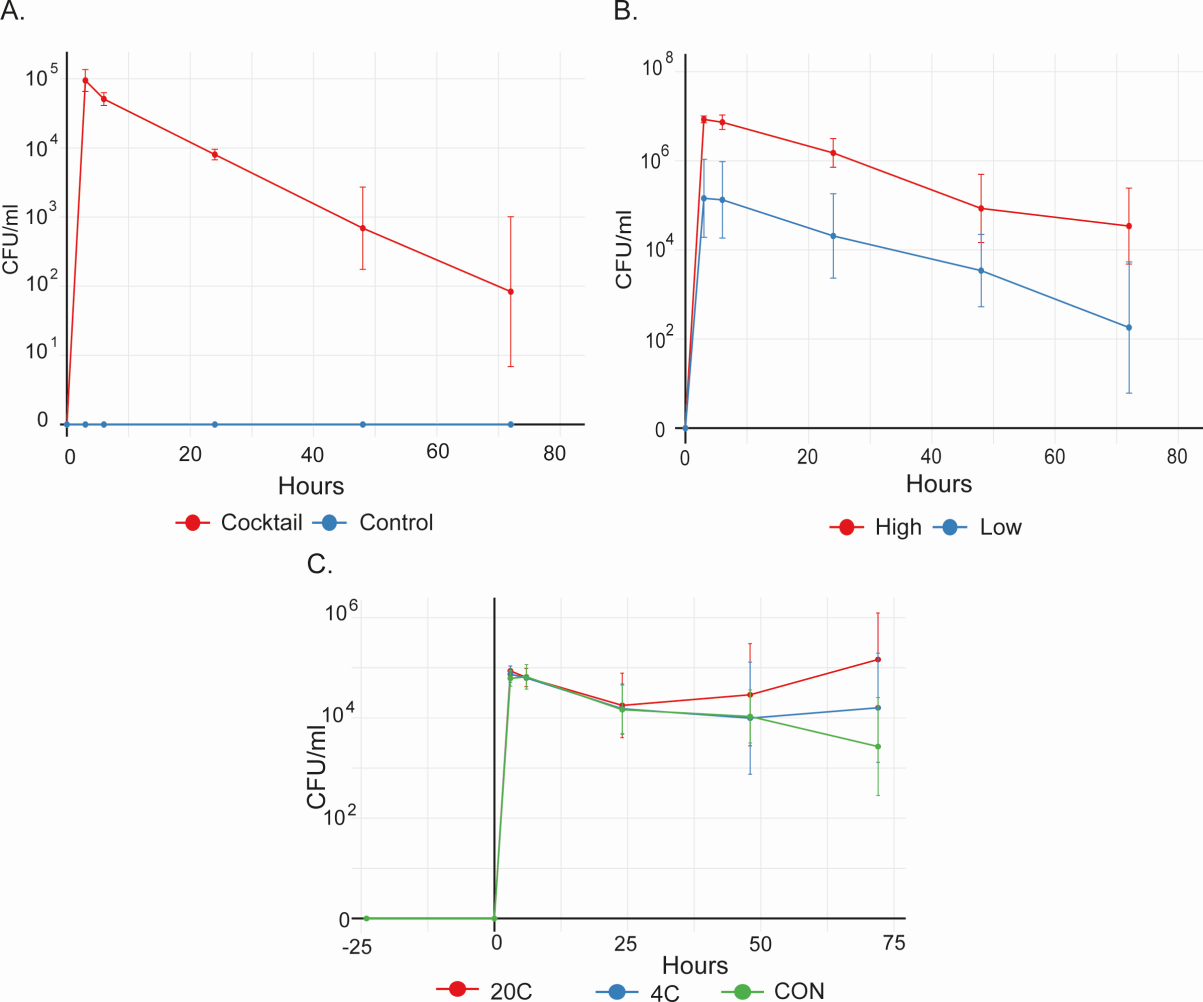
Effect of dose and ceftiofur on the recovery of *E. coli* cocktail bacteria (CFU/mL). A 17-isolate *E. coli* cocktail was added to a continuous flow vessel inoculated with chicken cecal contents. Bacteria were recovered following serial dilution and growth on Gassner media supplemented with rifampicin (50 µg/mL) and ceftiofur (4 µg/mL). (**A**) First experiment—vessels were dosed with 10^8^ CFU *E. coli* cocktail or sterile phosphate-buffered saline (PBS) (control), *N* = 6 vessels each. (**B**) Second experiment—vessels were dosed with 10^10^ CFU (high dose) or 10^8^ CFU (low dose) *E. coli* cocktail, *N* = 6 vessels each. (**C**) Third experiment—vessels were treated with ceftiofur at 20 µg/mL (20C), 4 µg/mL (4C), or PBS (no antibiotic control, CON) 24 h before, immediately prior to, and 24 h after dosing with *E. coli* cocktail (10^8^ CFU), *N* = 4 vessels each.

**Fig 3:**
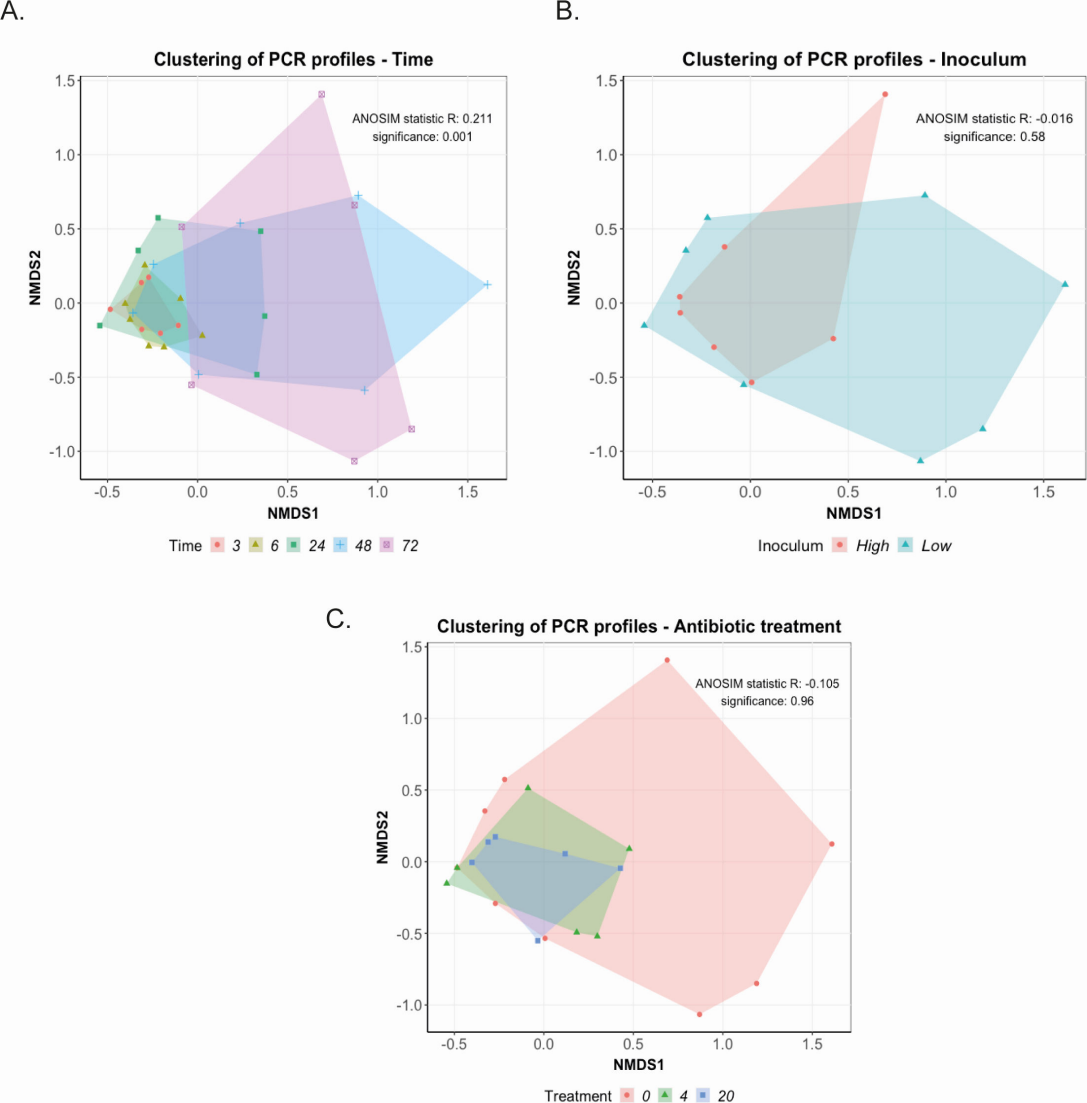
Non-metric multidimensional scaling (NMDS) plots showing changes in *E. coli* cocktail composition in the chicken cecal fermentation model. Continuous flow vessels containing chicken cecal contents were inoculated with a 17-member *E. coli* cocktail, and changes in the cocktail composition were determined using a PCR-based analysis following DNA extraction of bacterial lawns recovered on Gassner media supplemented with rifampicin (50 µg/mL) and ceftiofur (4 µg/mL). PCR presence or absence data were analyzed by NMDS to show changes in composition: (**A**) over time at 3, 6, 24, 48, and 72 h post-inoculation, (**B**) at high (10^10^ CFU) or low (10^8^ CFU) doses of the cocktail, and (**C**) following the addition of ceftiofur (at 0, 4, or 20 µg/mL). ANOSIM was used to test for statistical differences between groups. *N* = 4 to 6 biological replicates per group.

We previously described a panel of 17 individual ESBL-producing *E. coli* isolates, hereafter referred to as the *E. coli* cocktail, isolated from a range of hosts and each phylogenetically classified as associated with a particular host (30, Table 1). Herein, rifampicin-resistant (RIF^R^) mutants of the *E. coli* cocktail (10^8^ total CFU) were simultaneously introduced into the chicken cecal fermentation model to assess the ability of individual isolates to persist. Using selective media, we found that the concentration of total bacteria and *Enterobacteriaceae* recovered on LB and Gassner agar, respectively, remained relatively constant in three different experiments (Items S2A and S2B), while the mean concentration of the ceftiofur-resistant (CTF^R^) bacteria decreased by 2–3 logs (Item S2C). The concentration of *E. coli* cocktail bacteria (identified based on CTF^R^ and RIF^R^) similarly declined (Fig. 2A) with isolates only appearing to stabilize in vessel 4 (V4) between 48 and 72 h post-inoculation (Item S3A). No CTF^R^ organisms were recovered from control vessels that did not receive the *E. coli* cocktail. This indicated that most immigrant *E. coli* isolates were unable to be stably maintained in the cecal fermentation model. In our second experiment, increasing the inoculum dose to 10^10^ total CFU *E. coli* cocktail bacteria led to the recovery of approximately 2 log higher concentrations of CTF^R^ bacteria over time (Fig. 2B; Item S2C), with similar increases in the recovery of total bacteria and *Enterobacteriaceae* (Item S2A and S2B). Notably, concentrations of *E. coli* cocktail bacteria now appeared to stabilize in half of the vessels (H3, H4, and H6), with concentrations plateauing by 48 and 72 h (Item S3B). The higher concentration of CTF^R^ bacteria compared to *E. coli* cocktail bacteria in some vessels (H3 and H4, Item S3B) was suggestive of ESBL plasmid transmission to native bacteria. Finally, the addition of both low and high concentrations of CTF increased the recovery of immigrant *E. coli* over time (Fig. 3C), while the number of total bacteria and *Enterobacteriaceae* remained highest in control vessels that did not receive any antibiotics (Items S2A and S2B).

**TABLE 1:** Individual characteristics of the 17-member *E. coli* cocktail used in this study^*c*^

Isolate ID^*a*^	Source	Country of origin	Health status	Year	Phylogroup^*b*^	Sequence type (ST)	Resistance genes
Chicken1	Chicken	Vietnam	Healthy	2013	F	ST1163	*aadA5*, *bla*_CTX-M-27_, *bla*_EC-5_, *bla*_TEM-1_, *dfrA17*, *erm*(B), *mph*(A), *sul1*, *tet*(B)
Human1	Human	UK	Disease	2017	B2	ST131	*bla*_CTX-M-15_, *bla*_EC-5_
Human2	Human	UK	Disease	2017	B2	ST636	*aadA1, bla*_CTX-M-15_, *bla*_EC-19_, *dfrA1*, *sat2_gen*
Cattle1	Cattle	Germany	Disease	2016	D	ST362	*aac (3)-IVa, aph (4)-Ia, bla*_CTX-M-1_, *bla*_EC-8_, *dfrA36*, *floR*, *mph*(A), *sul2*, *tet*(Y)
Cattle2	Cattle	Germany	Healthy	2015	A	ST361	*aac (3)-IId, aac(6')-Ib-D181Y, aadA5, bla*_CTX-M-15_, *bla*_EC_*, bla*_OXA-1_*, bla*_TEM-1_, *dfrA17, tet*(B)
Cattle3	Cattle	Germany	Disease	2007	A	ST10	*aph(3')-Ia, aph(3'')-Ib, aph (6)-Id, bla*_CTX-M-1_, *bla*_EC_, *bla*_TEM-1_*, mph*(A), *sul2*, *tet*(B)
Cattle4	Cattle	Germany	Disease	2014	C	ST88	*bla*_CTX-M-1,_ *bla*_EC-13_, *mph*(A)
Cattle5	Cattle	Germany	Disease	2005	B1	ST448	*bla*_CTX-M-1_, *bla*_EC-13_, *mph*(A)
Cattle7	Cattle	Germany	Healthy	2011	F	ST648	*aac (3)-IId, aadA5*, *bla*_CTX-M-15_, *bla*_EC-19_, *bla*_CTX-M-15_, *dfrA17*, *mph*(A), *sul1*, *tet*(B)
Pig1	Pig	Germany	Disease	2016	A	ST361	*bla*_CTX-M-15,_ *bla*_EC_
Pig2	Pig	Germany	Disease	2011	A	ST206	*aac (3)-VIa, aadA2, aadA22, aph(3')-Ia, bla*_CTX-M-2_, *bla*_EC_, *bla*_TEM-1_, *lnu*(F), *qnrS1*, *sul1*, *tet*(A)
Pig3	Pig	Germany	Healthy	2011	C	ST410	*aac (3)-IId, aac(6')-Ib-D181Y, aadA5*, *aph(3'')-Ib*, *aph (6)-Id*, *bla*_CTX-M-15_, *bla*_CTX-M-172_, *bla*_EC-15_, *bla*_OXA-M-1_, *bla*_TEM-1_, *dfrA17*, *mph*(A), *sul1*, *sul*2, *tet*(B)
Pig4	Pig	Germany	Healthy	2016	B1	ST641	*aph(3'')-Ib, aph (6)-Id, bla*_CTX-M-1_, *bla*_EC-13_, *tet*(B)
Pig5	Pig	Germany	Healthy	2016	B1	ST2067	*bla*_CTX-M-1_, *bla*_EC-18_
Generalist4	Chicken	Spain	Healthy	2016	D	ST1011	*aac (3)-IId, aadA2, dfrA12, bla*_EC-15_, *bla*_TEM-143_, *mph*(A), *sul1*, *tet*(A)
Generalist3	Human	Germany	Disease	2009	A	ST12717	*bla*_EC_, *bla*_CTX-M-1_
Generalist1	Chicken	Vietnam	Healthy	2013	B1	ST162	*aac (3)-IId*, *aac (3)-Iva*, *aadA2, aadA5*, *aph(3'')-Ib*, *aph (4)-Ia*, *aph (6)-Id*, *bla*_CTX-M-55_, *bla*_EC-18_, *bla*_TEM-1_, *dfrA17*, *floR*, *mph*(A), *sul1*, *sul2*, *sul3*, *tet*(A)

^*a*^ Independent of the actual source of isolation, the isolate ID was assigned to reflect the host specificity score calculated for the respective isolate as explained in Materials and Methods.

^*b*^ Phylogroup predictions were reported elsewhere (18) and determined from the sequence data using ClemonTyper v1.4.1.

^*c*^ Additional information about the isolates used in the cocktail can be found in Item S12 in the supplemental material.

### Maintenance of immigrant *E. coli* isolates in the chicken cecal fermentation model was independent of designated host association

Having established parameters facilitating transient maintenance of immigrant isolates in the chicken cecal fermentation model, we sought to quantify the survival of individual members within the *E. coli* cocktail. Using a PCR-based approach (31), we found that *E. coli* associated with cattle (isolates cattle2 and cattle7) and pigs (isolate pig1) were maintained in at least half of the vessels over the 72 h incubation period (Item S4). Conversely, we found that some isolates were eliminated by 72 h (human2, cattle1, and pig3) or failed to establish in the vessels (generalist4). The single chicken-associated isolate (chicken1) did not appear to survive better than other host-associated isolates and was undetectable in most vessels by 72 h. Similar patterns were seen when the total number of bacteria in the *E. coli* cocktail was increased to 10^10^ total CFU and, for some isolates (chicken1, cattle3, cattle7, pig3, pig4, and generalist1), this change in concentration led to an increase in the number of vessels in which they could be recovered (Item S5). Interestingly, the addition of CTF did not result in consistent overgrowth of the *E. coli* cocktail, as few isolates were retained in all vessels. The isolates chicken1 and human1 were exceptions to this. However, they were also retained in untreated control vessels, suggesting that this was a community- or isolate-mediated effect rather than due to the presence of the antibiotic. Similarly, isolates cattle1, cattle5, and pig1 were retained in a higher percentage of control vessels, while cattle4 was retained in a higher percentage of vessels treated with a low level of the antibiotic (Item S6). Overall, adding CTF had no clear effect on which isolates were able to persist and for how long.

The PCR data facilitated the analysis of the changes in the composition of the *E. coli* cocktail (absence/presence) over time using a non-metric multi-dimensional scaling (NMDS) model. Time had a significant effect on the profile of isolates present in the cecal fermentation model (*P* = 0.001, Fig. 3A), with *E. coli* communities becoming more dissimilar as the incubation progressed. Isolate profiles at the start of the incubation differed from those at 72 h, where there was also greater variation in the vessels. However, analysis of NMDS plots showed that there was no significant effect of inoculum dose or antibiotic treatment on cocktail composition (*P* > 0.05, Fig. 3B and C), suggesting that the divergence of the *E. coli* isolate composition in each vessel is most likely due to uncharacterized inter- or intra-species interactions.

### Immigrant isolates were able to transfer ESBL plasmids to native *E. coli*

**Fig 4:**
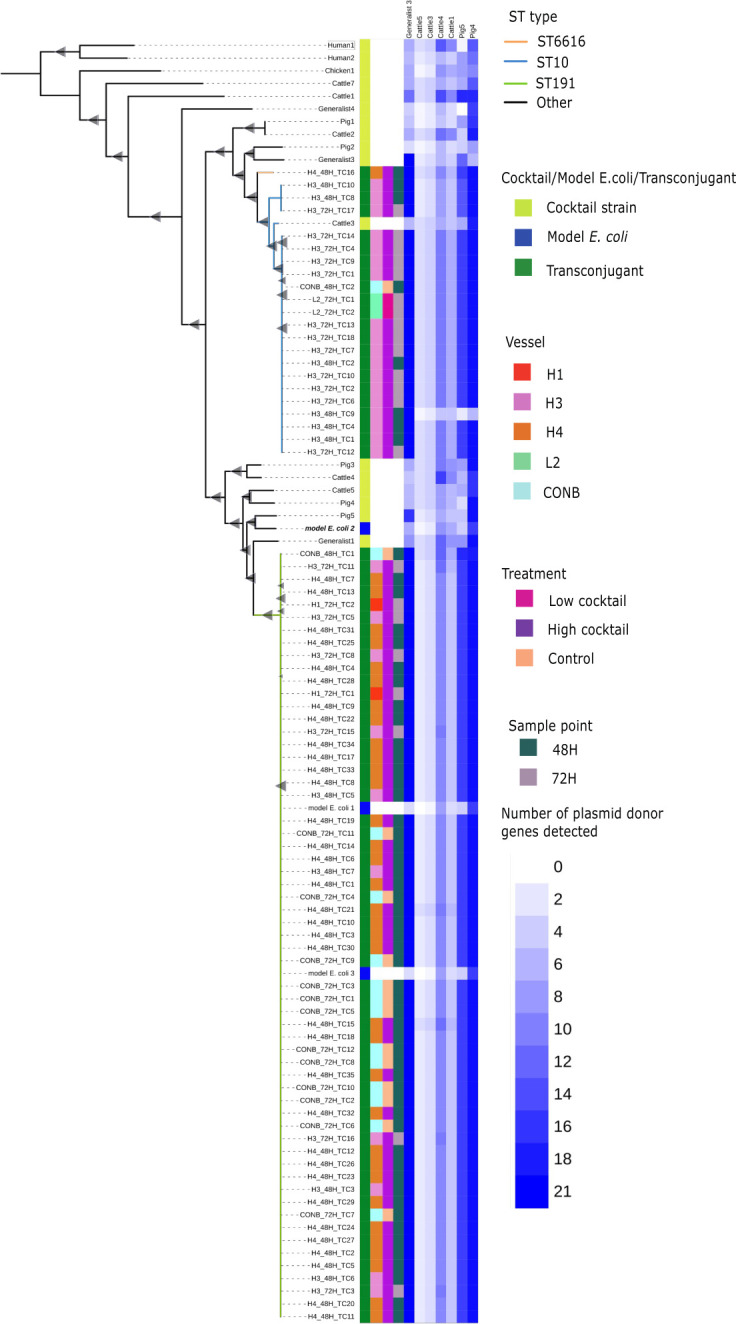
Phylogenetic relationship of resistant *E. coli*, three native *E. coli* present in the chicken ceca fermentation model, and the *E. coli* cocktail isolates. Continuous flow vessels containing chicken cecal contents were inoculated with a 17-member ESBL-producing *E. coli* cocktail in different experiments. Bacteria that were ceftiofur-resistant (CTF^R^), but rifampicin-sensitive (RIF^S^), were subject to whole-genome sequencing. Abricate was used to generate resistance gene profiles that led to seven members of the *E. coli* cocktail being identified as putative donors. The heatmap shows the number of genes in seven potential donor isolates that were identical to the 10-gene region on either side of *bla*_CTX-M-1_ in the putative transconjugant (score out of 21). Key: ST sequence type; Vessel H (20 µg/mL ceftiofur), L (4 µg/mL ceftiofur), C (no antibiotic control); treatment: high dose cocktail, low dose cocktail, control; sample point time post-inoculation.

As previously mentioned, the difference in CFU counts of CTF^R^ and CTF^R^ RIF^R^ microorganisms was indicative of ESBL plasmid transfer to native bacteria. Thus, we used replica plating and short-read whole-genome sequencing to determine the genotype of recipient *E. coli* and the likely origin of the plasmid. A total of 81 putative transconjugants were recovered from the following vessels over the three experiments: 10 from H3 at 48 h, 35 from H4 at 48 h, 2 from H1 at 72 h, 18 from H3 at 72 h, 2 from L2 at 72 h (from the second experiment), 2 from CON B at 48 h and 12 from CON B at 72 h (from the third experiment). In addition, three native *E. coli* isolated from chicken cecal content before cocktail addition were included in the analyses. Sequencing confirmed that transconjugants were *E. coli* belonging to sequence type (ST) 191 (61 isolates), ST10 (21 isolates), and ST6616 (1 isolate) (Item S7). Phylogenetic reconstruction of the 17 isolates from the *E. coli* cocktail, 3 native *E. coli* recovered from the fermentation mix, and 81 transconjugants showed that transconjugants were phylogenetically distinct from donor isolates (Fig. 4).

A total of 11 AMR genes, with *bla*_CTX-M-1_ being the most common, were identified in the potential transconjugants. Three combinations of resistance genes within the transconjugants persisted at higher frequencies and were recovered from multiple different vessels: (i) *aadA1*, *aadA2*, *bla*_CTX-M-1_, *bla*_EC_*bla*_TEM-1_, *cmlA1*, *Inu*(G), and *sul3* (17 of 81 isolates), (ii) *aadA22*, *bla*_CTX-M-1_, *bla*_EC-18_, *Inu*(G), and *tet*(B) (47 of 81 isolates), and (iii) *aadA22*, *bla*_CTX-M-1_, *bla*_EC-18_, *bla*_TEM-1,_ Inu(G), and *tet*(B) (13 of 81 isolates). There were four other isolates that did not fall into these three distinct groups; three of these formed another small clonal group, and the last (H4 48H TC16) was not genetically similar to any other isolate.

We noted that the *bla*_CTX-M-1_ gene was found in all transconjugants but absent from the native *E. coli* present in the cecal content used to seed the models, indicating that this was a marker of plasmid transfer. To identify which member(s) of the *E. coli* cocktail had shared their plasmid, the up- and down-stream regions adjacent to this locus were compared in the cocktail isolates that harbored *bla*_CTX-M-1_. Specifically, a custom BLAST database of the 10 genes immediately up- and down-stream of *bla*_CTX-M-1_ was compiled using generalist3, cattle1, cattle3, cattle4, cattle5, pig4, and pig5 sequences. All 21 genes associated with generalist3 were identified in all transconjugants (Fig. 4), implicating this isolate as the most likely donor for most transconjugants. While a high proportion of genes present in isolate pig4 were also detected in the transconjugants (20 out of 21 genes), alignment of the *bla*_CTX-M-1_ regions in pig4, generalist3, and a representative transconjugant revealed that only three genes immediately adjacent to *bla*_CTX-M-1_ were identical with the remaining 16 genes encoded elsewhere in the genome (Item S8). Additionally, these genes were also found in most other *E. coli* we sequenced, including model *E. coli* ST191 recipients initially isolated from cecal contents.

## DISCUSSION

Although the digestive tract of vertebrates often harbors multiple isolates of the same species at the same time (32), few studies have attempted to compare their relative persistence or ability to contribute to AMR transmission. Here, employing a chicken cecal fermentation model, we were able to track the dynamics of multiple ESBL-producing *E. coli* isolates residing simultaneously in a complex community and provide data identifying the ESBL-harboring isolate(s) most likely to have contributed to AMR transmission to native *E. coli* residing in the community.

Our data indicated that not all immigrant *E. coli* isolates persisted to the same extent. In our experiments, some pig- and cattle-associated isolates were recovered for longer periods than those associated with chicken or those lacking specific host-associated traits (generalists). Given that equivalent numbers of cells were added to each isolate, these data demonstrate that for some of these naturally occurring isolates, host exposure is less likely to result in establishment in the intestine (4, 13). In addition, it appears that host association, determined for these isolates through a combined analysis of their phylogeny and source metadata (18), did not necessarily restrict isolates associated with other hosts from establishing in the chicken cecum (19) or guarantee an ability for chicken-associated isolates to re-establish in the same host species. Interestingly, isolates belonging to phylogroups A (cattle2, pig1) and F (cattle7) were among those most often recovered, consistent with findings from healthy adult volunteers (4, 13).

The factors that govern residency remain poorly understood. Colonization resistance (33), a phenomenon conferred by the native microbiota on immigrant bacteria (34), is widely acknowledged. In addition, studies describing the properties of resident isolates can offer a glimpse into the attributes that may be important for successful immigrant isolates (4). Yet despite this knowledge, our ability to predict which immigrant isolates take up residence is limited. Determining this would go a long way to support a fuller understanding of zoonotic potential, transmission risk, and exploitation of probiotic therapies.

Dosing the vessels with ceftiofur helped to maintain immigrant populations, yet this did not result in consistent outgrowth of all resistant bacteria, challenging the assumption that resistant isolates typically outcompete susceptible isolates in the presence of a selective agent (35). It is possible that the acquisition of the ESBL-encoding plasmid reduced the fitness of the putative transconjugants (36, 37), a phenotype that can be exacerbated in the presence of a community (38). For example, a 20% reduction in bacterial growth rates was attributed to loss of the porins OmpC/OmpF following ESBL plasmid acquisition in *E. coli* (39). Whether this explains our inability to recover putative transconjugants from vessel H4 at 72 h, despite finding a large number (35 of 81 recovered in total) at earlier time points, is not known.

In addition to community effects on isolate fitness, it is possible that the presence of other microorganisms affected how members of the *E. coli* cocktail responded to antibiotics. Recent studies using both simple (two species [40]) and more complex (gut microbiotas [41]) microbial communities found that the growth and evolution of AMR in immigrant lineages were suppressed when other organisms were present. There is now a growing body of evidence that interspecies interactions can alter responses to antibiotics, and as such, the ecological context in which AMR is found must be considered when designing effective antibiotic stewardship criteria for both clinical and natural settings (42).

Finally, our data suggest that ESBL plasmid acquisition by native *E. coli* residing in the community occurred largely from a single representative, generalist3, a non-host-associated *E. coli* belonging to phylogroup A. Before we assessed the ability of isolates to conjugate in the chicken ceca model, we tested all isolates in individual mating experiments. This showed that all cocktail isolates had the ability to transfer their ESBL genes, conferring beta-lactam resistance to another *E. coli* in *vitro* (data not shown here). Surprisingly, generalist3 was not the most persistent cocktail isolate recovered in vessels where newly resistant isolates were detected. However, it should be noted that our data do not allow the absolute levels (CFU/g) of individual isolates to be determined, only their presence or absence. Thus, it is possible that this strain persisted at high densities during the initial stages of incubation before declining below detection limits. There is also a possibility that other gene transfer mechanisms occurred. For example, two of the three resistance gene profile groups mentioned previously included both *bla*_CTX-M-1_ and *bla*_TEM-1_, raising the possibility of transposase-mediated transfer of *bla*_TEM-1_ (43) by one of the six isolates that encoded both genes. It is also possible that significant fitness costs were associated with the uptake of other plasmids, leading to our inability to detect them within the cecal community. The gene *bla*_CTX-M-1_ has been identified as the most common ESBL gene found in livestock in Germany (44), the country of origin of generalist3. Found on highly transferable IncI1 or IncN plasmids (45, 46), transmission between individual *E. coli* isolates that can colonize animals or humans has been implied following detection of IncI1 *bla*_CTX-M-1_ plasmids in poultry, chicken meat, and human isolates in the Netherlands (47). Collectively, while our study demonstrates the complexities of understanding individual isolate dynamics in mixed-species communities, further studies are necessary to better understand AMR transmission (48).

The chicken cecal fermentation model we have used to observe ESBL transfer *in vitro* suffers from several limitations. Firstly, it is unlikely that the fermentation vessels contain all the taxonomic diversity present in the ceca due to the loss of strict anaerobes during inoculum preparation and the absence of “re-seeding” due to the transfer of feed particles and microorganisms from the small intestine. Secondly, we have chosen to use a pool of cecal content from 20 chickens to initiate our models instead of content from single birds. This ensured that we had enough cecal content to run all experiments, and this pool could be used to bring uniformity to the bacterial populations so that any effects seen were due to differing experimental conditions (e.g., different cocktail concentrations). We appreciate that this removed the intra-individual variation seen in the chicken cecal microbiota *in vivo*. Thirdly, there is no host involvement, as it is known that microorganisms within the gut respond to host-derived compounds, metabolites, and cells. Finally, while the fermentation vessels support a continuous flow of material, the cyclical emptying seen in live animals is not reproduced. Regardless, this study highlighted that plasmid transfer events between transient and resident *E. coli* can be observed in a complex microbiota *in vitro*. The molecular basis and multiple drivers of bacterial AMR transmission and evolution are difficult to study, but moving beyond a focus on single-species systems using *in vitro* models will help unveil these mechanisms.

## MATERIALS AND METHODS

### Continuous flow chicken cecal fermentation model

A modified continuous flow model originally described by Card et al. was used in this study (30). The model system consisted of six individual, unconnected fermentation vessels. Temperature (held at 42°C by a circulating water bath; Optima) and pH (kept within the range of 5.8–6.0 by Mettler-Toledo pH probes and Fermac 260 pH measurement and control modules) were automatically controlled (49). Media, provided via a multi-channel, variable-speed peristaltic pump (Electrolab) at a rate of 1.25 mL/h, supplied the vessels. All vessels and media reservoirs were under constant stirring using magnetic fleas and kept under anaerobic conditions by a continuous flow of anaerobic gas mixture (80% N_2_, 10% CO_2_, 10% H_2_) directly into the vessels and fresh media reservoir. Prior to each run, vessels (18 mL) and reservoirs (1 L) were filled with sterile Viande-Levure culture medium (10 g/L tryptone, 5 g/L NaCl, 5 g/L yeast extract, 2.5 g/L glucose, 2.4 g/L beef extract, 0.6 g/L cysteine.HCl) and left to gas anaerobically overnight.

Collection of cecal contents from chickens was approved by the University of Surrey’s animal ethics committee (NERA-2017-010-SVM). Twenty commercial broiler chickens were euthanized by cervical dislocation, and cecal contents were aseptically collected. Cecal samples were either used immediately as fermentation inoculum or frozen at −80°C for the generation of pooled inoculum. For this, samples from 15 individual animals were subsequently defrosted, combined, manually homogenized, and dispersed into 1 g aliquots for storage at −80°C for later use. Microbial communities were established by adding 2 mL of chicken cecal content slurry (1:1, cecal content:phosphate-buffered saline [PBS]) to each vessel, followed by batch culture for 24 h (no media flow) and 4 days of fermentation (with media flow) to allow stabilization of bacterial communities.

### Chicken cecal fermentation model experiments

#### Inoculum validation experiment

Fresh or frozen, individual or pooled samples of chicken cecal content were assessed as an inoculum source. Duplicate vessels were inoculated with either fresh or frozen cecal material from two chickens or with content pooled from both chickens (Item S9). After 24 h of batch culture and 4 days of fermentation (with media flow), samples were collected, and 16S rRNA gene profiles of microbial communities from the vessels were compared to those of chicken cecal content (see DNA extraction and sequencing for genomic analyses section below).

#### Selection of isolates in the *E. coli* “cocktail”

A defined cocktail of 17 host-associated ESBL-producing isolates was generated from a previously described collection of *E. coli* isolates (18). Host association was determined by using a library of 1,198 *E. coli* isolates collected in Germany, Spain, the UK, and Vietnam. One hundred fifty phylogenetic trees (bootstrap replicates) were generated, based on the alignment of the core genome of all isolates. For each tree and taking source into account, a host specificity score ranging from 0 to 1 was assigned to represent the extent to which a particular isolate was clustered with other isolates of the same host. For each isolate and host species, the geometric mean over the values per tree was used. The geometric mean approach was chosen to ensure only isolates that consistently clustered with the same-host isolates across most phylogenetic reconstructions received a relatively high host specificity score. The generalist score (which may serve as an indicator of an isolate's ability to colonize multiple host species) was determined by calculating the geometric mean of the host specificity scores for cattle, human, swine, and chicken hosts. In order to select suitable isolates for inclusion in the cocktail, the following criteria were applied: (i) isolates belonged to *E. coli sensu stricto* (no cryptic clade isolates), (ii) isolates either had a relatively high host specificity score (the top 20% for that host), or if the isolate had no pronounced specificity scores for any host, it was predicted to be a generalist, (iii) isolates carried an ESBL gene, (iv) isolates contained as few known virulence genes as possible and had been isolated preferably from a clinically healthy host, and (v) all countries and time periods were equally represented in the final list. To facilitate recovery from complex communities, rifampicin-resistant mutants were generated and used (31).

#### Preparation of *E. coli* cocktail

Single colonies from each isolate were aseptically transferred to separate tubes containing 10 mL LB broth supplemented with 4 µg/mL CTF and 50 µg/mL RIF. After aerobic incubation with shaking for 16 h, cultures were diluted 1:50 into fresh LB broth and grown to an OD_600_ of 0.5 before a further 1:10 dilution into fresh LB broth. Thereafter, pre-determined volumes were used to achieve equivalent isolated cell numbers in the cocktail (Item S10). In the first experiment, where 10^8^ CFU *E. coli* cocktail bacteria were added to each vessel, the pre-determined culture volumes were centrifuged at 3,000 × *g* for 3 min, supernatants were removed, and each cell pellet was resuspended in 0.5 mL sterile PBS. All the resuspended culture volumes were combined, and the final inoculum volume was made up to 10 mL with the addition of 1.5 mL PBS. Each vessel received 100 µL of the final mixture, amounting to 10^8^ CFU per vessel (5.88 × 10^6^ CFU of each isolate). In the second experiment, where 10^10^ CFU *E. coli* cocktail bacteria were added to each vessel, triplicate volumes of each isolate were generated, and each pellet was resuspended in 50 µL PBS. Three sets of inocula were prepared, each containing 50 µL of each isolate and 150 µL PBS to bring the final volume to 1 mL. Each vessel received 1 mL of the final mix, amounting to 10^10^ CFU per vessel (5.88 × 10^8^ CFU of each isolate). One milliliter of fermentation media was removed from each vessel prior to cocktail addition.

#### *E. coli* cocktail experiments

To assess the relative persistence and conjugative ability of isolates in the *E. coli* cocktail, several fermentation experiments were performed. In the first experiment, vessels were inoculated with 10^8^ CFU *E. coli* cocktail or sterile PBS as a control. In the second experiment to explore the impact of inoculum dose, three vessels were inoculated with 10^8^ (low, L) or 10^10^ (high, H) CFU *E. coli* cocktail. Both experiments were performed in duplicate, providing a total of six replicate vessels in the first (C1-6 control and V1-6 *E. coli* cocktail) and in the second (L1-6 low dose and H1-6 high dose) experiment. In the third experiment, CTF at 4 or 20 µg/mL was added to two vessels, with two control vessels (CON) receiving sterile PBS. CTF was added to the vessels 24 h before, immediately before, and 24 h after the addition of the *E. coli* cocktail (10^8^ CFU) to mimic a 3-day dosing regimen. Duplicate experiments were performed, giving four replicate vessels of each condition: 4C A-D contained 4 µg/mL CTF, 20C A-D contained 20 µg/mL CTF, and CON A-D contained PBS only.

#### Culture-based analyses

In all experiments, samples were taken from the vessels at the following time points: immediately before the *E. coli* cocktail addition (0 h) and 3, 6, 24, 48, and 72 h post-inoculation. In addition, for the third experiment, a sample was taken immediately before the first dose of antibiotics (−24 h).

#### Bacterial enumeration

Samples were serially diluted and plated using the Miles and Misra method (50) onto LB agar (for determination of total aerobic bacteria), Gassner agar (for total *Enterobacteriaceae*), Gassner supplemented with 4 µg/mL CTF (for CTF^R^ *Enterobacteriaceae*), and Gassner supplemented with 50 µg/mL RIF and 4 µg/mL CTF (for *E. coli* cocktail isolates). Plates were incubated at 35°C for 18 h, counted, and the CFU/mL of each of the four groups of bacteria was calculated. Replica plating was performed to assess if any potential transconjugants were present in the vessels. Colonies from the Gassner agar plates supplemented with CTF were picked and transferred onto fresh Gassner agar plates with CTF and Gassner agar plates with RIF and CTF. Any colonies that did not grow on the RIF and CTF plates but did grow on the CTF plates were deemed to be putative transconjugants and stored at −80°C for further analyses.

#### Fitting CFU counts under sampling uncertainty

To make optimal use of all count data generated in the experiments, we estimated the most likely CFU concentration per sample while accounting for sampling uncertainty associated with individual CFU counts. As each sample was serially diluted and plated, we obtained, per sample, *n* replicate measurements *X_i_* (1 ≤ *i* ≤ *n*) of the CFU count in a certain volume *V_i_* (mL). The volume *V_i_* represents the effective volume sampled, including a correction for the dilution factor used. We assumed that the CFU count *X_i_* follows a Poisson distribution with mean $\mu_{i}=V_{i}∙C$, where *C* is the actual concentration of cocktail bacteria present in the sample (CFU/mL). The probability mass function, i.e., the chance to find *X_i_* CFU given the mean *μ_i_*, is


$$P_{\mu_{i}}\left(X_{i}\right)=\frac{\mu_{i}^{X_{i}}}{X_{i}!}\cdot e^{-\mu_{i}}.$$

The likelihood *L(C*) of the *n* observations is


$$L\left(C\right)=\prod_{i=1}^{n}P_{\mu_{i}}\left(X_{i}\right).$$

And the corresponding log-likelihood *l(C*) is given by


$$l\left(C\right)=\sum_{i=1}^{n}\log\left(P_{\mu_{i}}\left(X_{i}\right)\right)=\sum_{i=1}^{n}\log\left(\frac{\mu_{i}^{X_{i}}}{X_{i}!}\cdot e^{-\mu_{i}}\right)=\sum_{i=1}^{n}\left(X_{i}\cdot \log\mu_{i}-\mu_{i}\right)-\sum_{i=1}^{n}\log X_{i}!=\sum_{i=1}^{n}\left(X_{i}\cdot \log\left(V_{i}\cdot C\right)-V_{i}\cdot C\right)-\sum_{i=1}^{n}\log X_{i}!=\sum_{i=1}^{n}\left(X_{i}\cdot \log C-V_{i}\cdot C\right)-\sum_{i=1}^{n}\left(X_{i}\cdot \log V_{i}+\log X_{i}!\right).$$

The last summation does not depend on the concentration of *C* and can, therefore, be ignored:


$$l\left(C\right)=\log C\cdot \sum_{i=1}^{n}X_{i}-C\cdot \sum_{i=1}^{n}V_{i}.$$

Per sample, we estimated the most likely concentration $\hat{C}=\sum_{i=1}^{n}X_{i}/\sum_{i=1}^{n}V_{i}$ by maximizing this log-likelihood function. Confidence intervals were computed based on the likelihood ratio, i.e., encompassing all possible values of *C* where $l\left(C\right)\geq l\left(\hat{C}\right)-0.5∙Q\left(p;\chi_{1}^{2}\right)$, in which $Q\left(p;\chi_{1}^{2}\right)$ is the quantile function of the chi-square distribution with one degree of freedom, with *p* being the coverage of the desired confidence interval (e.g., 0.95 for a 95% confidence interval).

Through these calculations, the most likely concentration $\hat{C}$ was calculated per sample, based on all dilutions of that sample. Lower CFU counts, which are associated with higher sampling uncertainty, contribute less $\hat{C}$ than relatively high CFU counts (i.e., at lower dilutions). The total sampling uncertainty around $\hat{C}$ is reflected in its 95% confidence interval.

### Detection and analysis of individual members of the *E. coli* cocktail

#### PCR-based identification of *E. coli* isolates

Bacterial growth on Gassner agar supplemented with 50 µg/mL RIF and 4 µg/mL CTF was resuspended in 2 mL sterile PBS and heated at 95°C for 10 min to lyse the cells. DNA was subjected to a quadruplex ORFan gene multiplex PCR assay to assess the presence of the 17 cocktail isolates at each time point as previously described (31). Data were expressed as isolate presence or absence for each of the 17 isolates in each vessel, at each time point.

#### Non-metric multidimensional scaling of PCR profiles

PCR presence/absence data were analyzed using NMDS to evaluate whether the composition of the *E. coli* cocktail changed over time, in response to increasing dose, or in the presence of CTF. Entries where no *E. coli* cocktail isolates were detected by PCR were removed, as these did not provide any information on the community composition. Using the Jaccard index as the distance matrix, the NMDS analysis was run multiple times, increasing the number of dimensions from 2 to 10. Each run with 15 tries (iterative) converges on the best solution based on the lowest stress. Then, based on a threshold stress of 0.1 (a good representation of actual dissimilarities), k = 3 dimensions was selected. An analysis of similarities (ANOSIM) test was performed to test whether the heterogeneity found between groups was significantly larger than the heterogeneity found within the groups. Finally, convex hull plots were created for the first two dimensions, visualizing the clustering of the PCR profiles. The R package vegan (51) was used for all analyses, and ggplot2 (52) was used to generate the visualizations.

#### DNA extraction and sequencing for genomic analyses

Samples from the cecal fermentation model for DNA extraction were processed by centrifugation (11,337 × *g* for 10 min), the supernatant was discarded, and DNA was extracted from the cell pellet using the DNeasy Power Soil kit (QIAGEN) in accordance with the manufacturer’s protocol. Eluted DNA was resuspended in 100 µL of DNA-free water.

#### 16S rRNA gene sequencing

A total of 12 DNA extracts from the validation experiment, along with DNA extracted from three chicken cecal content samples, were sent for 16S rRNA gene sequencing on the MiSeq Illumina platform (Animal and Plant Health Agency, Weybridge, UK). The V4 and V5 regions of the 16S rRNA genes were amplified using the primers U515F (5′-GTGYCAGCMGCCGCGGTA-3′) and U927R (5′-CCCGYCAATTCMTTTRAGT-3′) (53). Amplification was performed using the following PCR conditions: 95°C for 3 min, 25 cycles of 95°C for 30 s, 55°C for 35 s, and 72°C for 1 min, followed by 72°C for 8 min. Amplicons were purified using Ampure XP magnetic beads (Beckman Coulter). Each sample was tagged with a unique pair of indices and sequencing primers using Nextera XT v2 Index kits and 2× Kalpa HiFi HotStart ReadyMix. The PCR conditions used for this were: 95°C for 30 s, 55°C for 30 s, 72°C for 30 s, and 72°C for 5 min. The concentration of each sample was quantified using the Quantiflour assay (Promega), and concentrations were normalized before pooling all samples. Sequencing was performed on an Illumina MiSeq 2 × 300 bp (Illumina, Cambridge, UK).

16S rRNA gene sequencing files were processed and analyzed using QIIME2 (qiime2-2018.4) (53). All sequence files were imported and converted into a single QIIME2 data file (qiime tools import). DADA2 (54) was used to denoise and remove low-quality reads at positions 20 and 280. Alignment was performed on the sequences (qiime alignment mafft), and this alignment was masked to remove positions that were highly variable (qiime alignment mask). FastTree was used to generate a phylogenetic tree from the masked alignment (qiime phylogeny fastree), and midpoint rooting was applied (qiime phylogeny midpoint-root). The reference database Greengenes (version 2018) (55) was utilized and trained on the sequences generated from the study (qiime feature-classifier classify-sklearn). Taxonomic composition of all samples and samples by groups was generated (qiime taxa barplot).

#### Whole-genome sequencing

DNA was extracted from the 17 *E. coli* cocktail isolates using the Promega Wizard DNA extraction kit (Wisconsin, USA) according to the manufacturer’s instructions. Genomic DNA libraries were prepared using Nextera XT Library Prep Kit (Illumina, San Diego, USA) with the following modifications: input DNA was increased twofold, and PCR elongation time was increased to 45 s. DNA quantification and library preparation were carried out on a Hamilton Microlab Star automated liquid handling system (Hamilton Bonaduz AG, Switzerland). Libraries were sequenced at MicrobesNG (Birmingham, UK) on Illumina NovaSeq 6000 using a 250 bp paired-end protocol. Sequence adapters were trimmed using Trimmomatic (version 0.30) with a sliding cut-off of Q15 and a sliding window of 5 base pairs. *De novo* assembly was performed using SPAdes V3.7 (56). Contig files were uploaded to the University of Surrey’s remote Linux server and the following analyses carried out: MLST (v2.17.0) (57) to identify the sequence types (ST) of the transconjugants, ParSNP (v1.6) (58) to generate a phylogenetic tree (using auto-picked reference genome of CONB72HTC7), Abricate (v1.0.0) (58) to identify resistance genes (using the NCBI database, release 245) and plasmids (using the PlasmidFinder database) (v2.0.1). Sequencing files from the 16S rRNA gene amplicon sequencing of cecal content and fermentation liquid, along with the whole-genome sequencing of trans-conjugant isolates, can be accessed on the NCBI Sequence Read Archive under BioProject ID PRJNA926526. Some of the genomes of the cocktail isolates were uploaded previously to the NCBI Sequencing Read Archive, and a table including all cocktail isolates, their Bioproject and SRA accession numbers can be found in Item S12.

## SUPPLEMENTAL MATERIAL

Supplemental material aem.00822-25-s0001.pdf Items S1 to S12.

ASM does not own the copyrights to Supplemental Material that may be linked to, or accessed through, an article. The authors have granted ASM a non-exclusive, world-wide license to publish the Supplemental Material files. Please contact the corresponding author directly for reuse.

## References

[1] Ercumen A, Pickering AJ, Kwong LH, Arnold BF, Parvez SM, Alam M, Sen D, Islam S, Kullmann C, Chase C, Ahmed R, Unicomb L, Luby SP, Colford JM Jr. 2017. Animal feces contribute to domestic fecal contamination: evidence from E. coli measured in water, hands food, flies, and soil in Bangladesh. Environ Sci Technol 51:8725–8734. doi:10.1596/31369 28686435 PMC5541329

[2] Zhang C, Derrien M, Levenez F, Brazeilles R, Ballal SA, Kim J, Degivry M-C, Quéré G, Garault P, van Hylckama Vlieg JET, Garrett WS, Doré J, Veiga P. 2016. Ecological robustness of the gut microbiota in response to ingestion of transient food-borne microbes. ISME J 10:2235–2245. doi:10.1038/ismej.2016.13 26953599 PMC4989305

[3] Martínez I, Muller CE, Walter J. 2013. Long-term temporal analysis of the human fecal microbiota revealed a stable core of dominant bacterial species. PLoS One 8:e69621. doi:10.1371/journal.pone.0069621 23874976 PMC3712949

[4] Martinson JNV, Pinkham NV, Peters GW, Cho H, Heng J, Rauch M, Broadaway SC, Walk ST. 2019. Rethinking gut microbiome residency and the Enterobacteriaceae in healthy human adults. ISME J 13:2306–2318. doi:10.1038/s41396-019-0435-7 31089259 PMC6776003

[5] Kim W, Racimo F, Schluter J, Levy SB, Foster KR. 2014. Importance of positioning for microbial evolution. Proc Natl Acad Sci USA 111:E1639–E1647. doi:10.1073/pnas.1323632111 24715732 PMC4000849

[6] Conway T, Cohen PS. 2015. Commensal and pathogenic Escherichia coli metabolism in the gut. Microbiol Spectr 3. doi:10.1128/microbiolspec.MBP-0006-2014 PMC4510460 26185077

[7] Delmas J, Gibold L, Faïs T, Batista S, Leremboure M, Sinel C, Vazeille E, Cattoir V, Buisson A, Barnich N, Dalmasso G, Bonnet R. 2019. Metabolic adaptation of adherent-invasive Escherichia coli to exposure to bile salts. Sci Rep 9:2175. doi:10.1038/s41598-019-38628-1 30778122 PMC6379400

[8] Niehus R, Oliveira NM, Li A, Fletcher AG, Foster KR. 2021. The evolution of strategy in bacterial warfare via the regulation of bacteriocins and antibiotics. Elife 10:e69756. doi:10.7554/eLife.69756 34488940 PMC8423443

[9] Ochman H, Lawrence JG, Groisman EA. 2000. Lateral gene transfer and the nature of bacterial innovation. Nature 405:299–304. doi:10.1038/35012500 10830951

[10] Bauer MA, Kainz K, Carmona-Gutierrez D, Madeo F. 2018. Microbial wars: competition in ecological niches and within the microbiome. Microb Cell 5:215–219. doi:10.15698/mic2018.05.628 29796386 PMC5961915

[11] Rakoff-Nahoum S, Foster KR, Comstock LE. 2016. The evolution of cooperation within the gut microbiota. Nature 533:255–259. doi:10.1038/nature17626 27111508 PMC4978124

[12] Carlson BA, Nightingale KK, Mason GL, Ruby JR, Choat WT, Loneragan GH, Smith GC, Sofos JN, Belk KE. 2009. Escherichia coli O157:H7 strains that persist in feedlot cattle are genetically related and demonstrate an enhanced ability to adhere to intestinal epithelial cells. Appl Environ Microbiol 75:5927–5937. doi:10.1128/AEM.00972-09 19617387 PMC2747851

[13] Martinson JNV, Walk ST. 2020. Escherichia coli residency in the gut of healthy human adults. EcoSal Plus 9. doi:10.1128/ecosalplus.ESP-0003-2020 PMC7523338 32978935

[14] Ewers C, Bethe A, Semmler T, Guenther S, Wieler LH. 2012. Extended-spectrum β-lactamase-producing and AmpC-producing Escherichia coli from livestock and companion animals, and their putative impact on public health: a global perspective. Clin Microbiol Infect 18:646–655. doi:10.1111/j.1469-0691.2012.03850.x 22519858

[15] Ludden C, Raven KE, Jamrozy D, Gouliouris T, Blane B, Coll F, de Goffau M, Naydenova P, Horner C, Hernandez-Garcia J, Wood P, Hadjirin N, Radakovic M, Brown NM, Holmes M, Parkhill J, Peacock SJ. 2019. One health genomic surveillance of Escherichia coli demonstrates distinct lineages and mobile genetic elements in isolates from humans versus livestock. mBio 10:e02693-18. doi:10.1128/mBio.02693-18 30670621 PMC6343043

[16] Nguyen VT, Jamrozy D, Matamoros S, Carrique-Mas JJ, Ho HM, Thai QH, Nguyen TNM, Wagenaar JA, Thwaites G, Parkhill J, Schultsz C, Ngo TH. 2019. Limited contribution of non-intensive chicken farming to ESBL-producing Escherichia coli colonization in humans in Vietnam: an epidemiological and genomic analysis. J Antimicrob Chemother 74:561–570. doi:10.1093/jac/dky506 30629197 PMC6376849

[17] Wu G, Day MJ, Mafura MT, Nunez-Garcia J, Fenner JJ, Sharma M, van Essen-Zandbergen A, Rodríguez I, Dierikx C, Kadlec K, Schink A-K, Wain J, Helmuth R, Guerra B, Schwarz S, Threlfall J, Woodward MJ, Woodford N, Coldham N, Mevius D. 2013. Comparative analysis of ESBL-positive Escherichia coli Isolates from animals and humans from the UK, the Netherlands and Germany. PLoS One 8:e75392. doi:10.1371/journal.pone.0075392 24086522 PMC3784421

[18] Tiwari SK, van der Putten BCL, Fuchs TM, Vinh TN, Bootsma M, Oldenkamp R, La Ragione R, Matamoros S, Hoa NT, Berens C, et al.. 2023. Genome-wide association reveals host-specific genomic traits in Escherichia coli. BMC Biol 21:76. doi:10.1186/s12915-023-01562-w 37038177 PMC10088187

[19] Zhi S, Li Q, Yasui Y, Edge T, Topp E, Neumann NF. 2015. Assessing host-specificity of Escherichia coli using a supervised learning logic-regression-based analysis of single nucleotide polymorphisms in intergenic regions. Mol Phylogenet Evol 92:72–81. doi:10.1016/j.ympev.2015.06.007 26115845

[20] D’Andrea MM, Arena F, Pallecchi L, Rossolini GM. 2013. CTX-M-type β-lactamases: a successful story of antibiotic resistance. Int J Med Microbiol 303:305–317. doi:10.1016/j.ijmm.2013.02.008 23490927

[21] Bezabih YM, Sabiiti W, Alamneh E, Bezabih A, Peterson GM, Bezabhe WM, Roujeinikova A. 2021. The global prevalence and trend of human intestinal carriage of ESBL-producing Escherichia coli in the community. J Antimicrob Chemother 76:22–29. doi:10.1093/jac/dkaa399 33305801

[22] Geser N, Stephan R, Kuhnert P, Zbinden R, Kaeppeli U, Cernela N, Haechler H. 2011. Fecal carriage of extended-spectrum β-lactamase-producing Enterobacteriaceae in swine and cattle at slaughter in Switzerland. J Food Prot 74:446–449. doi:10.4315/0362-028X.JFP-10-372 21375882

[23] Haenni M, Châtre P, Métayer V, Bour M, Signol E, Madec J-Y, Gay E. 2014. Comparative prevalence and characterization of ESBL-producing Enterobacteriaceae in dominant versus subdominant enteric flora in veal calves at slaughterhouse, France. Vet Microbiol 171:321–327. doi:10.1016/j.vetmic.2014.02.023 24629776

[24] Hiroi M, Yamazaki F, Harada T, Takahashi N, Iida N, Noda Y, Yagi M, Nishio T, Kanda T, Kawamori F, Sugiyama K, Masuda T, Hara-Kudo Y, Ohashi N. 2012. Prevalence of extended-spectrum β-lactamase-producing Escherichia coli and Klebsiella pneumoniae in food-producing animals. J Vet Med Sci 74:189–195. doi:10.1292/jvms.11-0372 21979457

[25] Liu X, Liu H, Wang L, Peng Q, Li Y, Zhou H, Li Q. 2018. Molecular characterization of extended-spectrum β-lactamase-producing multidrug resistant Escherichia coli from swine in northwest China. Front Microbiol 9:1756. doi:10.3389/fmicb.2018.01756 30123199 PMC6085443

[26] Sabia C, Stefani S, Messi P, de Niederhäusern S, Bondi M, Condò C, Iseppi R, Anacarso I. 2017. Extended-spectrrum beta-lactamase and plasmid-mediated AmpC genes in swine and ground pork. J Food Saf 37:1–5. doi:10.1111/jfs.12282

[27] Schmid A, Hörmansdorfer S, Messelhäusser U, Käsbohrer A, Sauter-Louis C, Mansfeld R. 2013. Prevalence of extended-spectrum β-lactamase-producing Escherichia coli on Bavarian dairy and beef cattle farms. Appl Environ Microbiol 79:3027–3032. doi:10.1128/AEM.00204-13 23455336 PMC3623142

[28] Lambrecht E, Van Coillie E, Van Meervenne E, Boon N, Heyndrickx M, Van de Wiele T. 2019. Commensal E. coli rapidly transfer antibiotic resistance genes to human intestinal microbiota in the Mucosal Simulator of the Human Intestinal Microbial Ecosystem (M-SHIME). Int J Food Microbiol 311:108357. doi:10.1016/j.ijfoodmicro.2019.108357 31536878

[29] Smet A, Rasschaert G, Martel A, Persoons D, Dewulf J, Butaye P, Catry B, Haesebrouck F, Herman L, Heyndrickx M. 2011. In situ ESBL conjugation from avian to human Escherichia coli during cefotaxime administration. J Appl Microbiol 110:541–549. doi:10.1111/j.1365-2672.2010.04907.x 21143712

[30] Card RM, Cawthraw SA, Nunez-Garcia J, Ellis RJ, Kay G, Pallen MJ, Woodward MJ, Anjum MF. 2017. An in vitro chicken gut model demonstrates transfer of a multidrug resistance plasmid from Salmonella to commensal Escherichia coli. mBio 8. doi:10.1128/mBio.00777-17 PMC5516254 28720731

[31] Ferrandis-Vila M, Tiwari SK, Mamerow S, Semmler T, Ferrandis-Vila M, Tiwari SK, van der Putten B, Trung NV, Oldenkamp R, Bootsma M, et al.. 2022. Using unique ORFan genes as strain-specific identifiers for Escherichia coli. BMC Microbiol 22:135. doi:10.1186/s12866-022-02508-y 35585491 PMC9118744

[32] Foster-Nyarko E, Pallen MJ. 2022. The microbial ecology of Escherichia coli in the vertebrate gut. FEMS Microbiol Rev 46:fuac008. doi:10.1093/femsre/fuac008 35134909 PMC9075585

[33] Lawley TD, Walker AW. 2013. Intestinal colonization resistance. Immunology 138:1–11. doi:10.1111/j.1365-2567.2012.03616.x 23240815 PMC3533696

[34] Khan I, Bai Y, Zha L, Ullah N, Ullah H, Shah SRH, Sun H, Zhang C. 2021. Mechanism of the gut microbiota colonization resistance and enteric pathogen infection. Front Cell Infect Microbiol 11:716299. doi:10.3389/fcimb.2021.716299 35004340 PMC8733563

[35] Sandegren L. 2014. Selection of antibiotic resistance at very low antibiotic concentrations. Ups J Med Sci 119:103–107. doi:10.3109/03009734.2014.904457 24694026 PMC4034545

[36] San Millan A, MacLean RC. 2017. Fitness costs of plasmids: a limit to plasmid transmission. Microbiol Spectr 5. doi:10.1128/microbiolspec.mtbp-0016-2017 PMC11687550 28944751

[37] San Millan A, Toll-Riera M, Qi Q, Betts A, Hopkinson RJ, McCullagh J, MacLean RC. 2018. Integrative analysis of fitness and metabolic effects of plasmids in Pseudomonas aeruginosa PAO1. ISME J 12:3014–3024. doi:10.1038/s41396-018-0224-8 30097663 PMC6246594

[38] Klümper U, Recker M, Zhang L, Yin X, Zhang T, Buckling A, Gaze WH. 2019. Selection for antimicrobial resistance is reduced when embedded in a natural microbial community. ISME J 13:2927–2937. doi:10.1038/s41396-019-0483-z 31384011 PMC6864104

[39] Adler M, Anjum M, Andersson DI, Sandegren L. 2013. Influence of acquired β-lactamases on the evolution of spontaneous carbapenem resistance in Escherichia coli. J Antimicrob Chemother 68:51–59. doi:10.1093/jac/dks368 22977158

[40] Nair RR, Andersson DI. 2023. Interspecies interaction reduces selection for antibiotic resistance in Escherichia coli. Commun Biol 6:331. doi:10.1038/s42003-023-04716-2 36973402 PMC10043022

[41] Baumgartner M, Bayer F, Pfrunder-Cardozo KR, Buckling A, Hall AR. 2020. Resident microbial communities inhibit growth and antibiotic-resistance evolution of Escherichia coli in human gut microbiome samples. PLoS Biol 18:e3000465. doi:10.1371/journal.pbio.3000465 32310938 PMC7192512

[42] Bottery MJ, Pitchford JW, Friman VP. 2021. Ecology and evolution of antimicrobial resistance in bacterial communities. ISME J 15:939–948. doi:10.1038/s41396-020-00832-7 33219299 PMC8115348

[43] Branger C, Ledda A, Billard-Pomares T, Doublet B, Fouteau S, Barbe V, Roche D, Cruveiller S, Médigue C, Castellanos M, Decré D, Drieux-Rouze L, Clermont O, Glodt J, Tenaillon O, Cloeckaert A, Arlet G, Denamur E. 2018. Extended-spectrum β-lactamase-encoding genes are spreading on a wide range of Escherichia coli plasmids existing prior to the use of third-generation cephalosporins. Microb Genom 4:e000203. doi:10.1099/mgen.0.000203 30080134 PMC6202452

[44] Irrgang A, Hammerl JA, Falgenhauer L, Guiral E, Schmoger S, Imirzalioglu C, Fischer J, Guerra B, Chakraborty T, Käsbohrer A. 2018. Diversity of CTX-M-1-producing E. coli from German food samples and genetic diversity of the bla_CTX-M-1_ region on IncI1 ST3 plasmids. Vet Microbiol 221:98–104. doi:10.1016/j.vetmic.2018.06.003 29981716

[45] Dahmen S, Haenni M, Madec JY. 2012. IncI1/ST3 plasmids contribute to the dissemination of the blaCTX-M-1 gene in Escherichia coli from several animal species in France. J Antimicrob Chemother 67:3011–3012. doi:10.1093/jac/dks308 22872449

[46] Moodley A, Guardabassi L. 2009. Transmission of IncN plasmids carrying blaCTX-M-1 between commensal Escherichia coli in pigs and farm workers. Antimicrob Agents Chemother 53:1709–1711. doi:10.1128/AAC.01014-08 19188380 PMC2663060

[47] Leverstein-van Hall MA, Dierikx CM, Cohen Stuart J, Voets GM, van den Munckhof MP, van Essen-Zandbergen A, Platteel T, Fluit AC, van de Sande-Bruinsma N, Scharinga J, Bonten MJM, Mevius DJ, National ESBL surveillance group. 2011. Dutch patients, retail chicken meat and poultry share the same ESBL genes, plasmids and strains. Clin Microbiol Infect 17:873–880. doi:10.1111/j.1469-0691.2011.03497.x 21463397

[48] De Wit G, Svet L, Lories B, Steenackers HP. 2022. Microbial interspecies interactions and their impact on the emergence and spread of antimicrobial resistance. Annu Rev Microbiol 76:179–192. doi:10.1146/annurev-micro-041320-031627 35609949

[49] Macfarlane GT, Englyst HN. 1986. Starch utilization by the human large intestinal microflora. J Appl Bacteriol 60:195–201. doi:10.1111/j.1365-2672.1986.tb01073.x 2423494

[50] Miles AA, Misra SS, Irwin JO. 1938. The estimation of the bactericidal power of the blood. J Hyg (Lond) 38:732–749. doi:10.1017/s002217240001158x 20475467 PMC2199673

[51] Blanchet F, Kindt R, Legendre P, Minchin P, Solymos P, Stevens M, Szoecs E, Wagner H, Barbour M, Bedward M, et al.. 2024. vegan: community ecology package, vR package version 2.7-0. https://github.com/vegandevs/vegan.

[52] Wickham H. 2016. ggplot2: elegant graphics for data analysis. Springer International Publishing.

[53] Bolyen E, Rideout JR, Dillon MR, Bokulich NA, Abnet CC, Al-Ghalith GA, Alexander H, Alm EJ, Arumugam M, Asnicar F, et al.. 2019. Reproducible, interactive, scalable and extensible microbiome data science using QIIME 2. Nat Biotechnol 37:852–857. doi:10.1038/s41587-019-0209-9 31341288 PMC7015180

[54] Callahan BJ, McMurdie PJ, Rosen MJ, Han AW, Johnson AJA, Holmes SP. 2016. DADA2: High-resolution sample inference from Illumina amplicon data. Nat Methods 13:581–583. doi:10.1038/nmeth.3869 27214047 PMC4927377

[55] DeSantis TZ, Hugenholtz P, Larsen N, Rojas M, Brodie EL, Keller K, Huber T, Dalevi D, Hu P, Andersen GL. 2006. Greengenes, a chimera-checked 16S rRNA gene database and workbench compatible with ARB. Appl Environ Microbiol 72:5069–5072. doi:10.1128/AEM.03006-05 16820507 PMC1489311

[56] Bankevich A, Nurk S, Antipov D, Gurevich AA, Dvorkin M, Kulikov AS, Lesin VM, Nikolenko SI, Pham S, Prjibelski AD, Pyshkin AV, Sirotkin AV, Vyahhi N, Tesler G, Alekseyev MA, Pevzner PA. 2012. SPAdes: a new genome assembly algorithm and its applications to single-cell sequencing. J Comput Biol 19:455–477. doi:10.1089/cmb.2012.0021 22506599 PMC3342519

[57] Jolley KA, Bray JE, Maiden MCJ. 2018. Open-access bacterial population genomics: BIGSdb software, the PubMLST.org website and their applications. Wellcome Open Res 3:124. doi:10.12688/wellcomeopenres.14826.1 30345391 PMC6192448

[58] Treangen TJ, Ondov BD, Koren S, Phillippy AM. 2014. The Harvest suite for rapid core-genome alignment and visualization of thousands of intraspecific microbial genomes. Genome Biol 15:524. doi:10.1186/s13059-014-0524-x 25410596 PMC4262987

