# Supplementary material for: Evidence of ESBL plasmid transfer and selective persistence of multiple host-associated Escherichia coli isolates in a chicken cecal fermentation model
Source: Appl Environ Microbiol. 2025 Sep 19;91(10):e00822-25. doi: 10.1128/aem.00822-25 (PMC12542655; doi:10.1128/aem.00822-25)
Supplement: Supplemental material — Items S1 to S12. [file aem.00822-25-s0001.pdf]

SUPPLEMENTARY MATERIAL

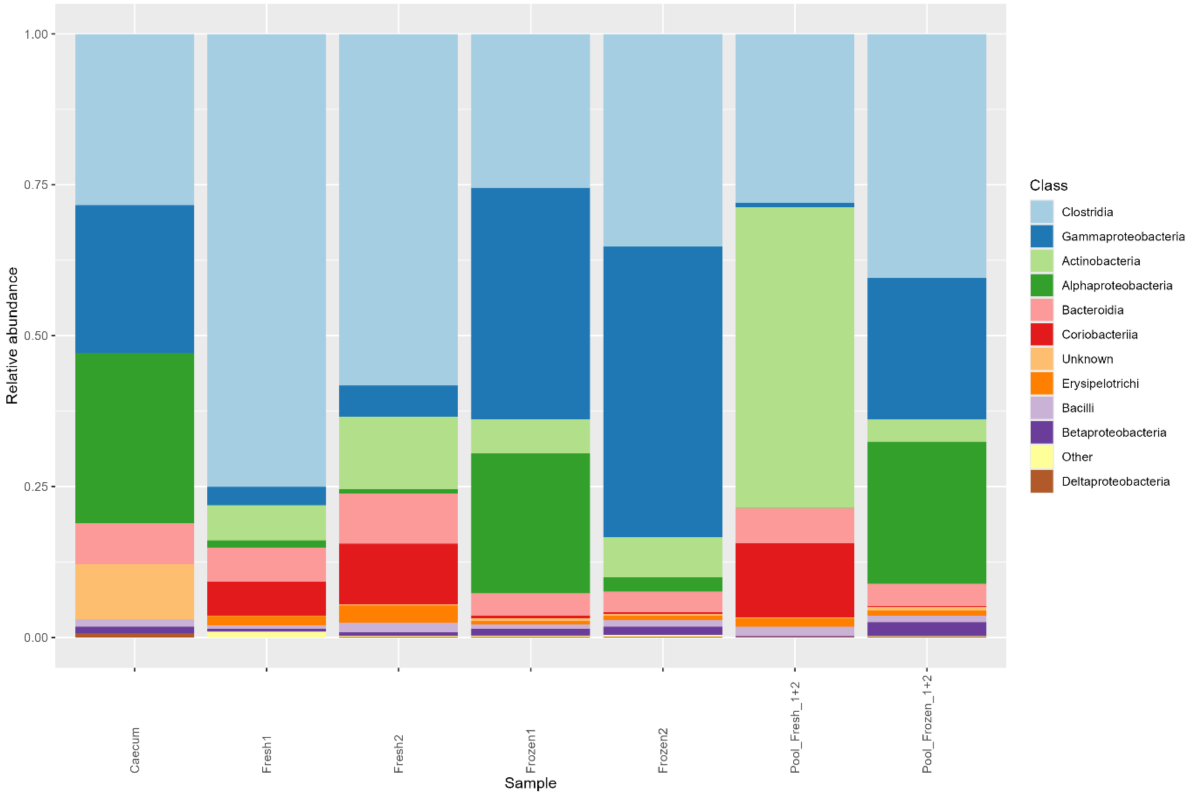

Supplementary Item 1: Bacterial class composition based on 16S rRNA gene sequencing of samples from the chicken caeca and from the model after four days of fermentation. Bars represent the mean percentage abundance for bacterial phyla found in the chicken caeca (N=3), two vessels inoculated with fresh caecal content from two independent chickens (Fresh1, Fresh2), two vessels inoculated with content Fresh1 and Fresh2 combined (Pool Fresh 1+2), vessels inoculated with caecal content from independent chickens, frozen at the time of collection and subsequently re-used (Frozen1 and Frozen2), and two vessels inoculated with a frozen pooled sample (Pool Frozen1+2). Duplicate vessels were run for each condition.

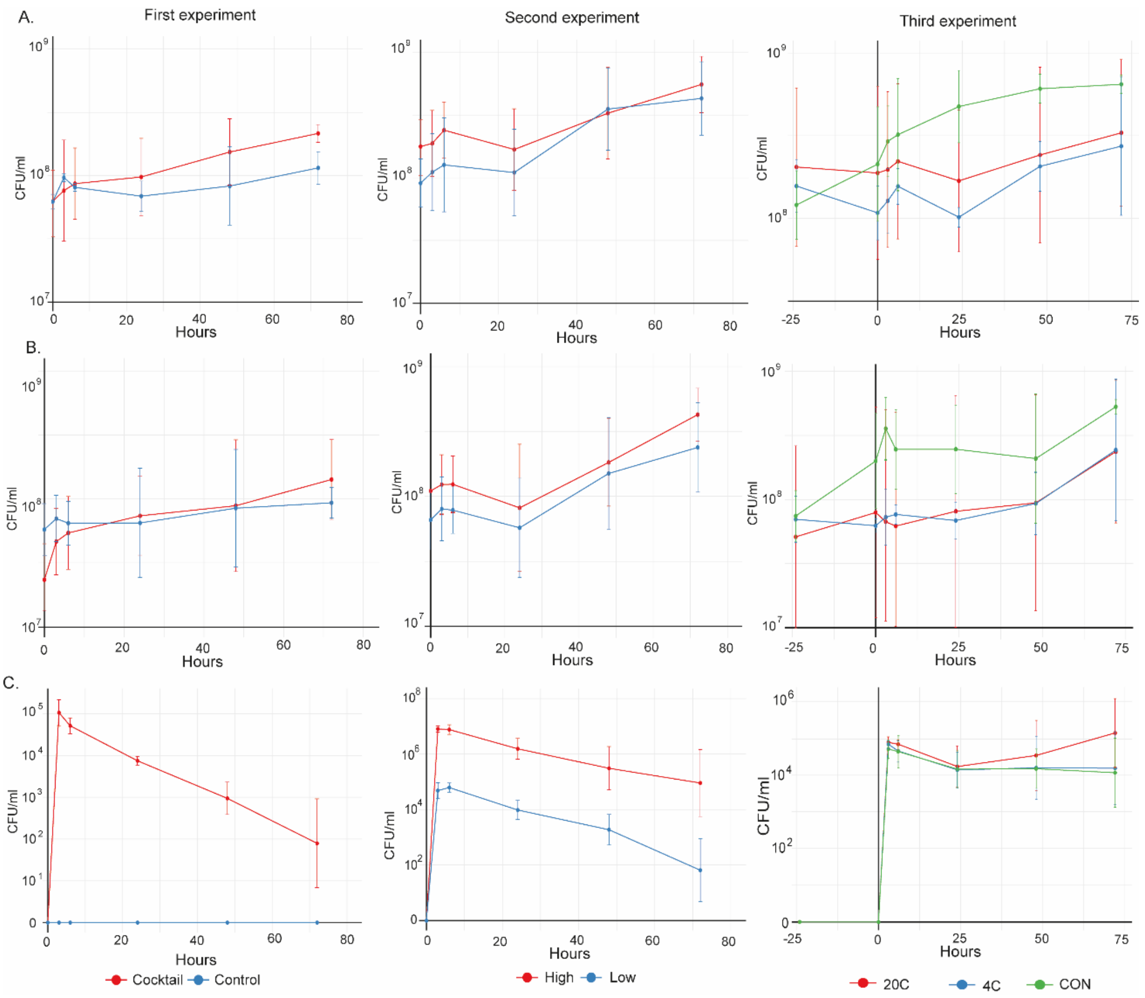

Supplementary Item 2: Concentration (CFU/ml) of (A) total bacteria, (B) Enterobacteriaceae and (C) ceftiofur-resistant bacteria recovered from an *in vitro* model of the chicken caeca. Continuous flow vessels containing chicken caecal contents were inoculated with a 17-member *E. coli* isolate cocktail and the recovery of bacterial populations determined over time. The groups of bacteria were: A) total bacteria, B) Enterobacteriaceae and C) CTF^R. High; high dose of the *E. coli* cocktail (10^{10} total bacteria) Low, low dose of the *E. coli* cocktail (10^8 total bacteria); 20C, 20 µg/ml ceftiofur; 4C, 4 µg/ml ceftiofur; CON, control.

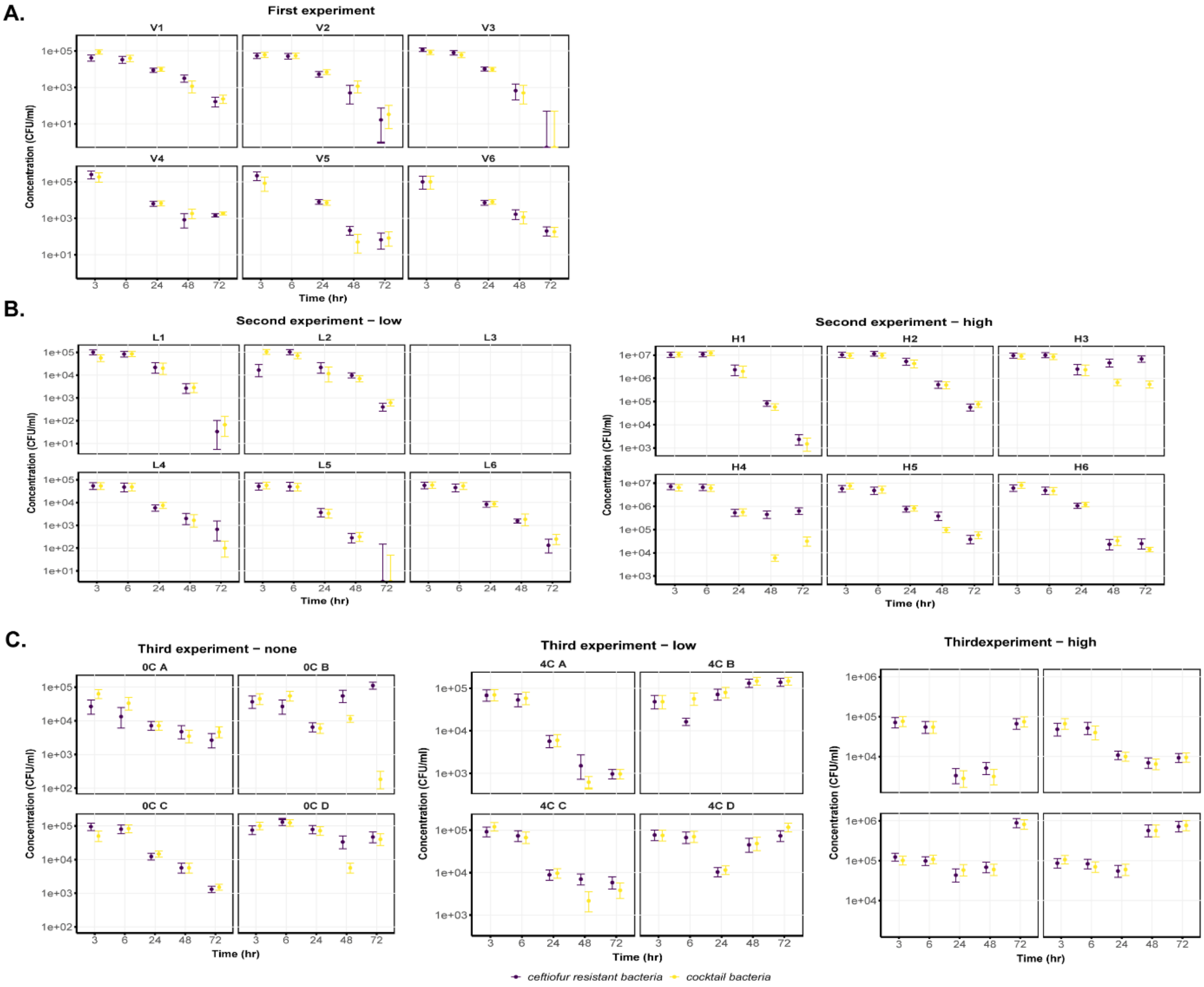

Supplementary Item 3: Concentration (CFU/ml) of ceftiofur-resistant bacteria and the *E. coli* cocktail isolates (ceftiofur- and rifampicin-resistant) from the three experiments. A) The vessels that received the *E. coli* cocktail in the first experiment (control vessels contained no ceftiofur- or rifampicin-resistant bacteria), B) the vessels innoculated with the high (10^{10} total bacterial) and low (10^{8} total bacteria) *E. coli* load cocktail in the second experiment and C) the vessels that received high (20 µg/ml) and low (4 µg/ml) concentrations of ceftiofur or no antibiotics in the third experiment (antibiotic). Error bars represent the 95 % confidence interval around the most likely value.

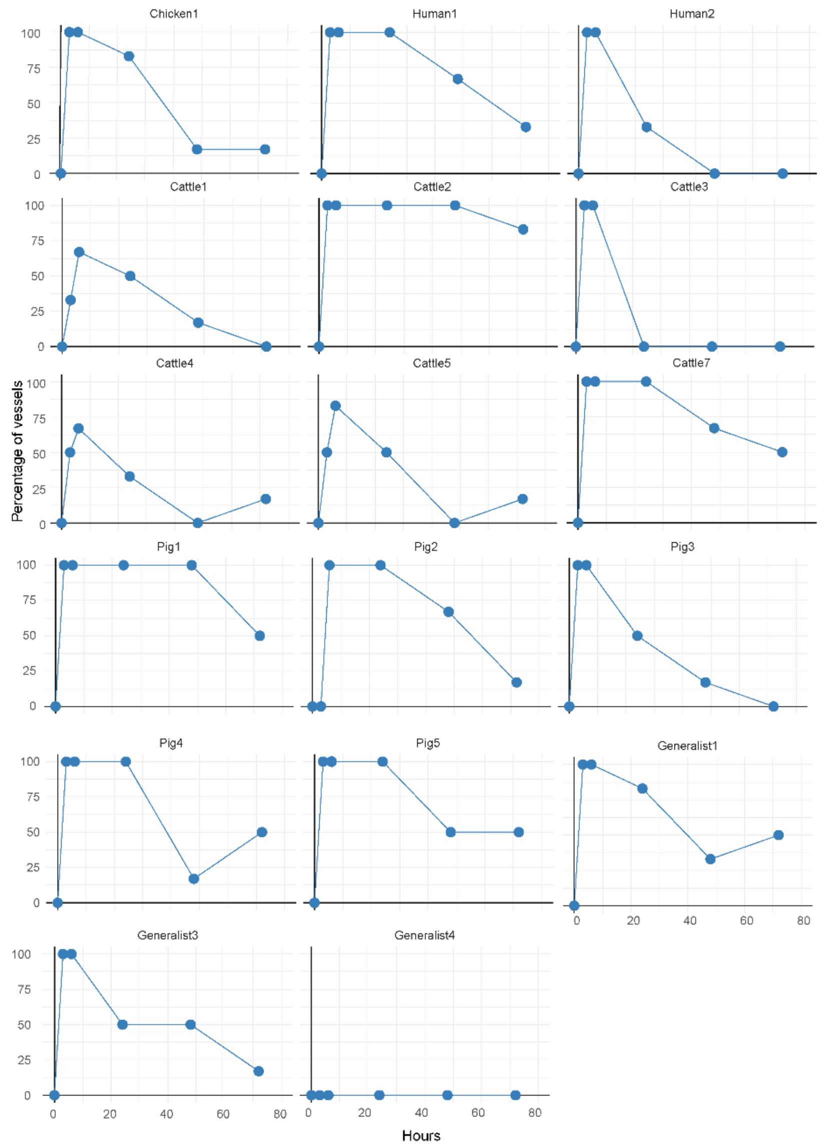

Supplementary item 4: The percentage of experimental vessels that contained each member of the 17-member *E. coli* cocktail recovered on selective plates followed by PCR multiplex assays. ***E. coli*** isolate presence or absence was determined using plate sweeps of colonies. Lines and data points showing the same values lie on top of each other and therefore only the line or point for the low vessels is visible. N= 6 vessels in each case.

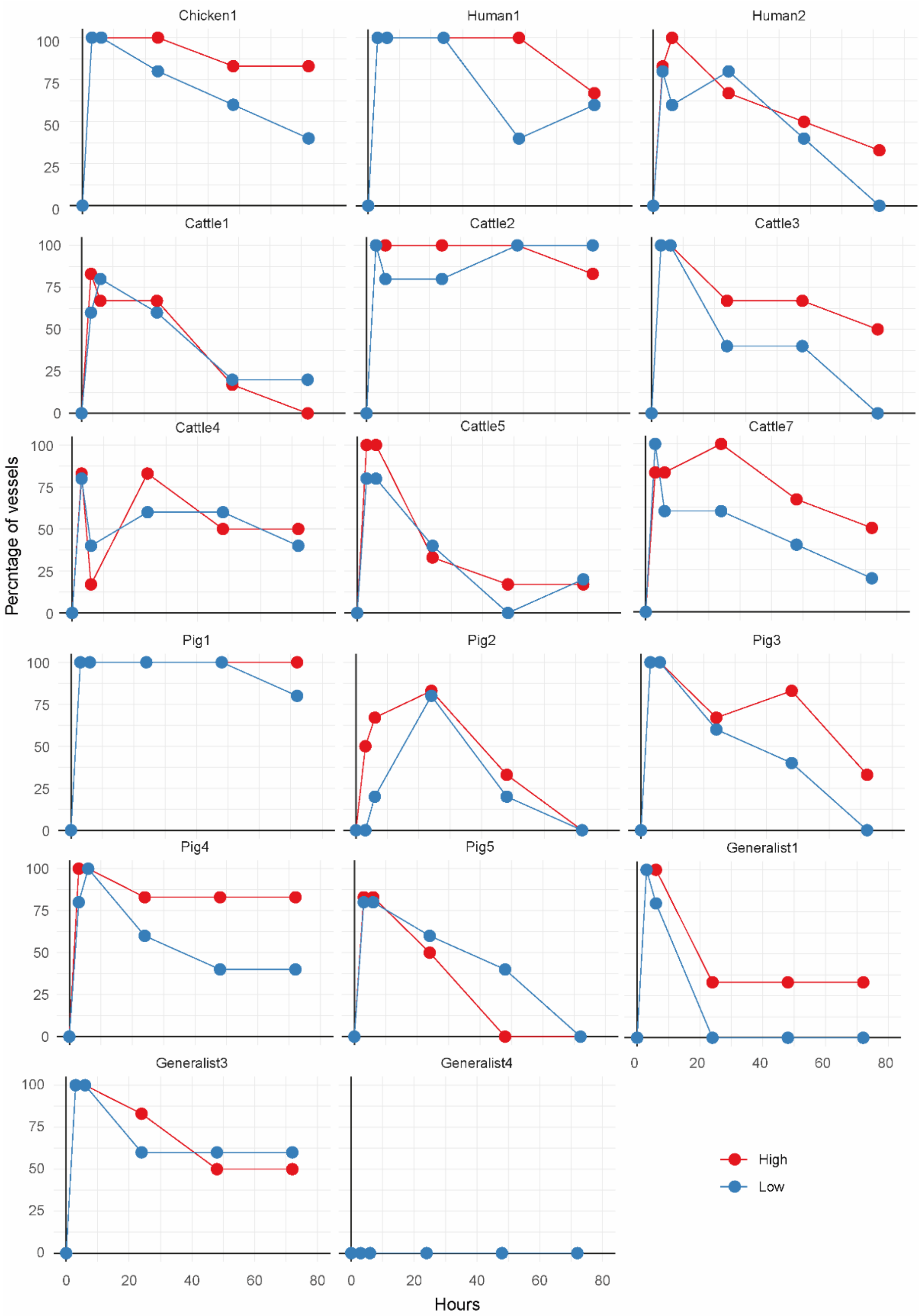

68 **Supplementary item 5: The percentage of experimental vessels that contained each member of**

69 **the 17-member *E. coli* cocktail when inoculated with high (10^{10} CFU) or low (10^{8} CFU) doses of**

70 **the cocktail.** Isolate presence or absence was determined using plate sweeps of colonies. Lines and

71 data points showing the same values lie on top of each other and therefore only the line or point for

72 the low vessels is visible. N=6 vessels in each case.

73

74

75

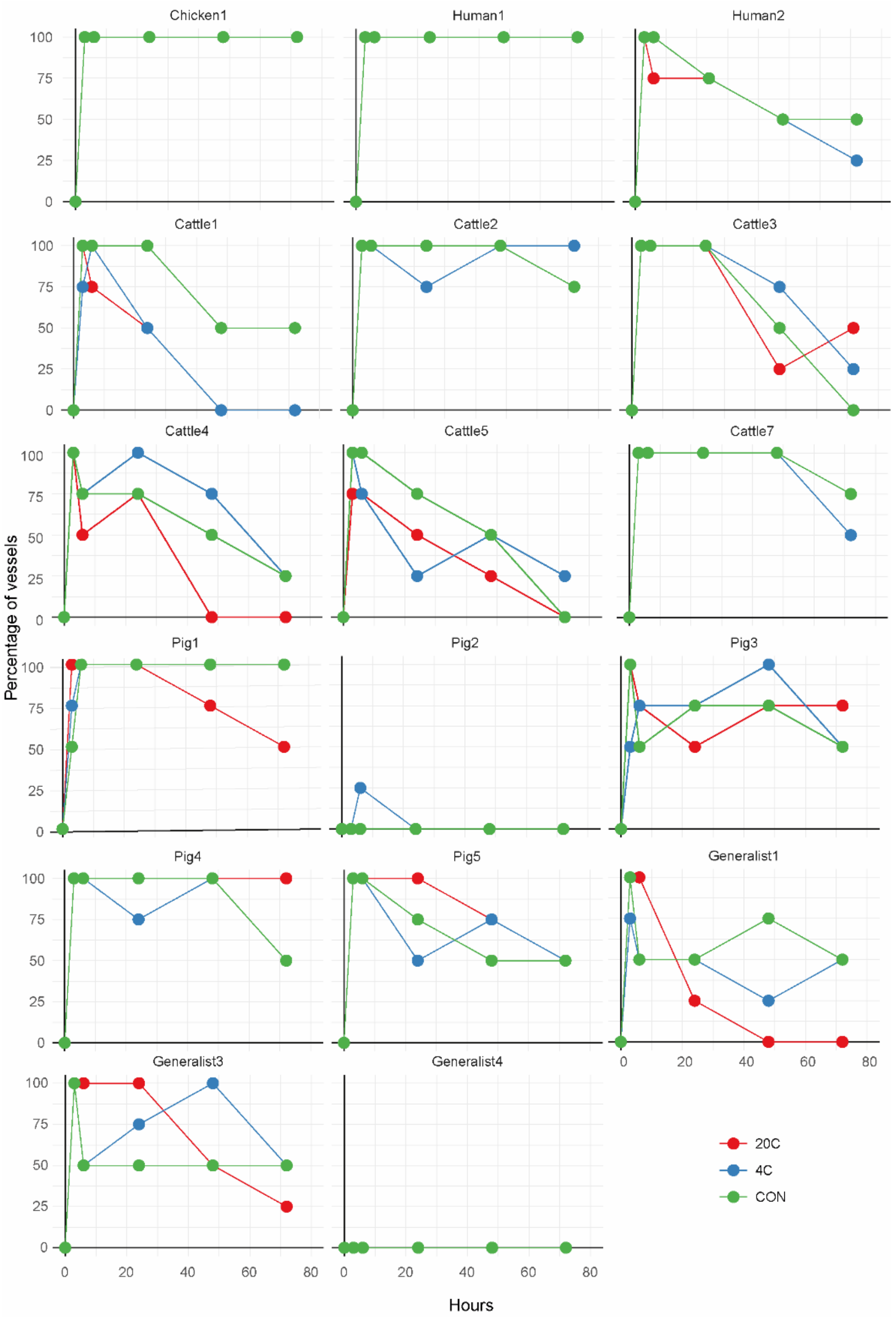

Supplementary item 6: The percentage of experimental vessels that contained each member of the 17-member *E. coli* cocktail when inoculated with 20 µg/ml CTF (20C), 4 µg/ml CTF (4C) or no antibiotic (CON). Isolate presence or absence was determined using plate sweeps of colonies. Lines and data points showing the same values lie on top of each other and therefore only the line or point for the control vessels is visible. N= 4 vessels in each case.

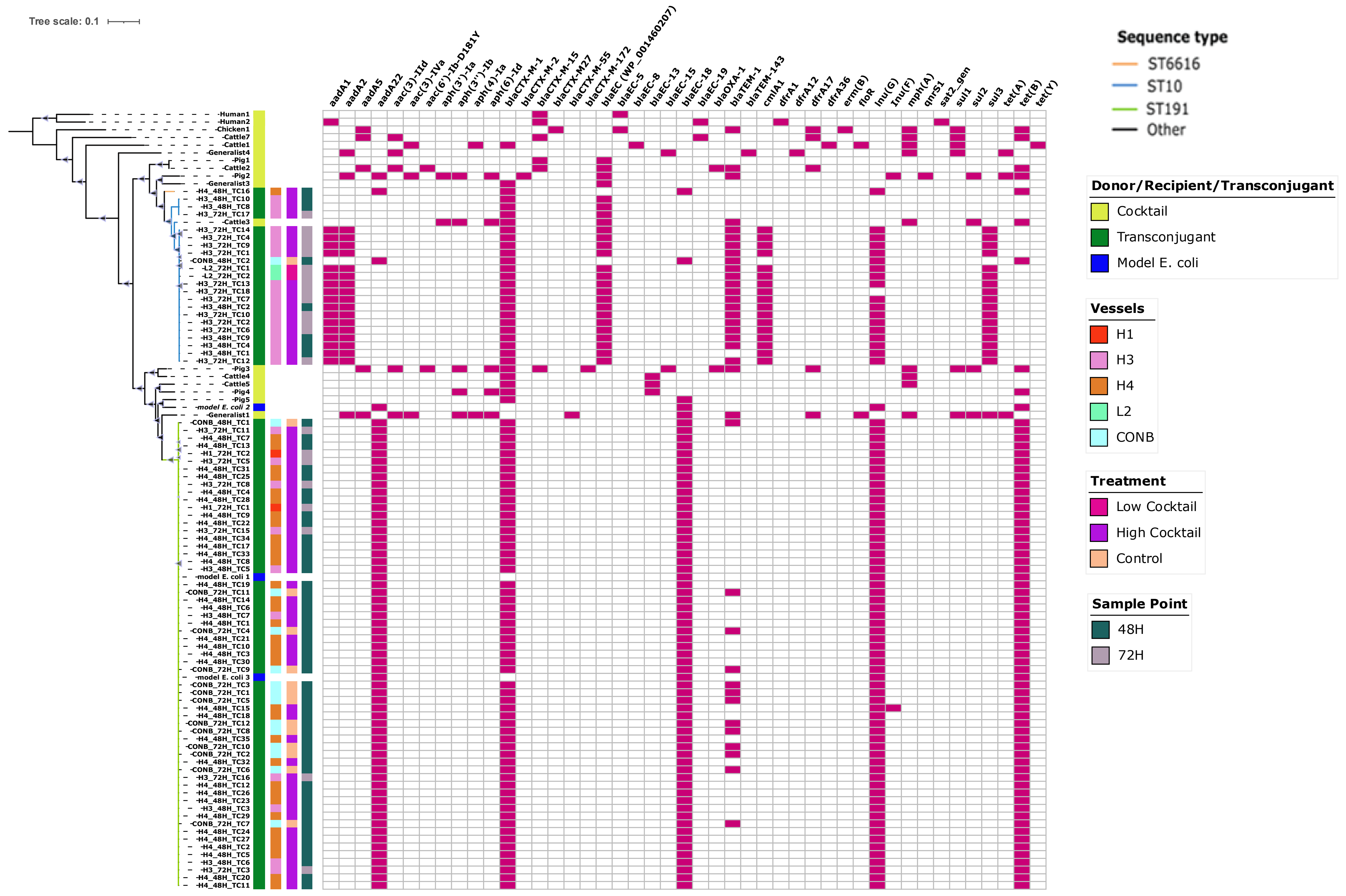

Supplementary item 7: Phylogenetic tree of all *E. coli* isolates subjected to whole genome sequencing. This included 17 *E. coli* isolates included in the cocktail, transconjugants and native *E. coli* showing the absence/presence of antibiotic resistance genes. Isolates were sequenced using Illumina sequencing and Abricate to identify putative resistance genes.

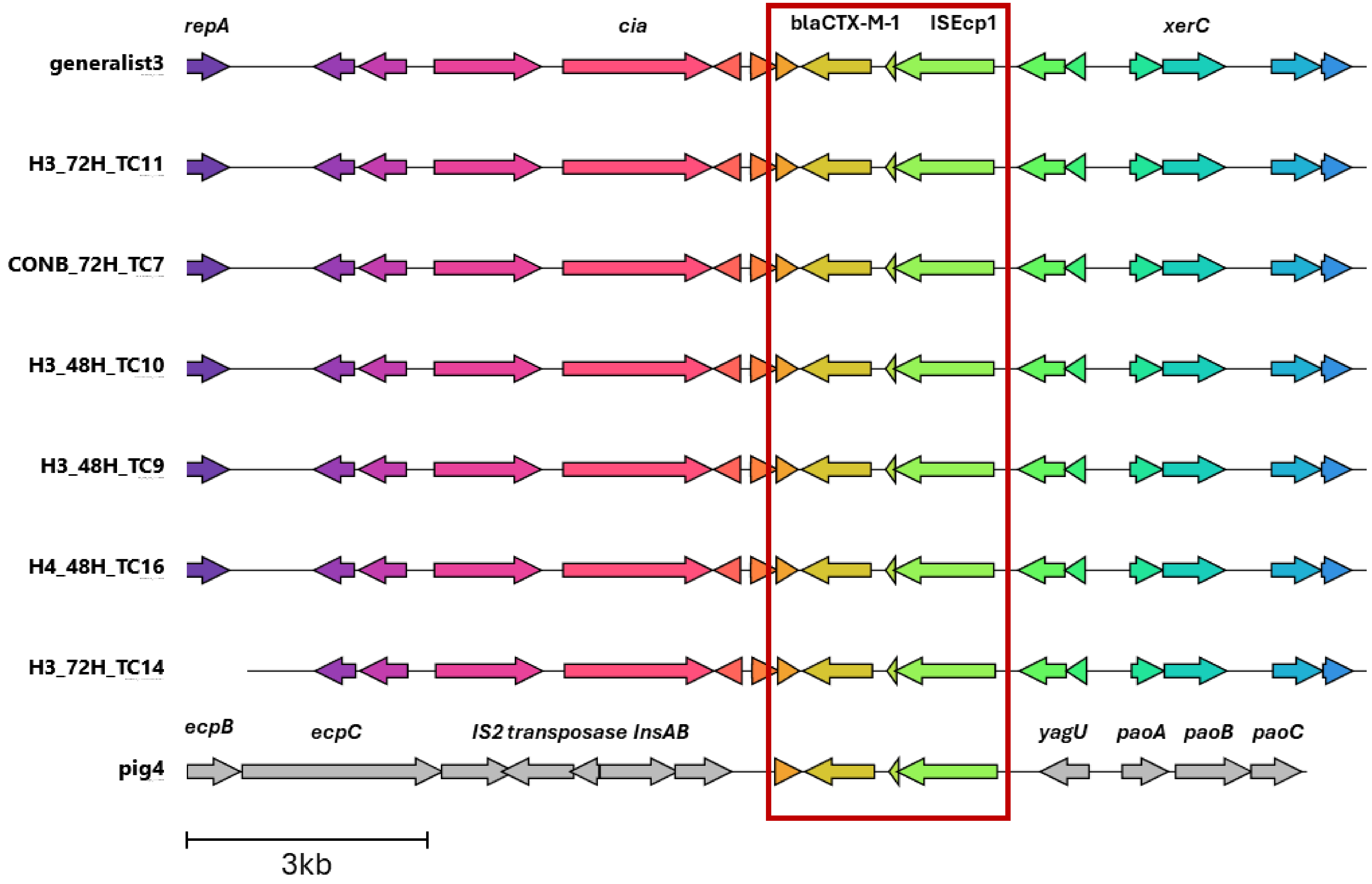

Supplementary Item 8: Schematic representation of the alignment of genes immediately upstream and downstream of the *bla*$_{CTX\text{-}M\text{-}1}$ gene. This gene was identified in the cocktail isolates generalist3, pig4, and representative transconjugants recovered from the *in vitro* gut model that are representative of the antimicrobial resistance gene (ARG) profiles observed (H3_72H_TC11, CONB_72H_TC7, H3_48H_TC10, H3_48H_TC9, H4_48H_TC16 and H3_72_TC14). All transconjugants show 100% identity with genes from generalist3 implicating generalist3 as the *bla*$_{CTX\text{-}M\text{-}1}$ donor. Whilst a high proportion of pig4-derived genes are also present in transconjugants, these are shown to be endogenously encoded by the model *E. coli* ST-191 natively present in the caecal community. The genes coloured grey in the schematic of pig4 code for hypothetical proteins of unknown function.

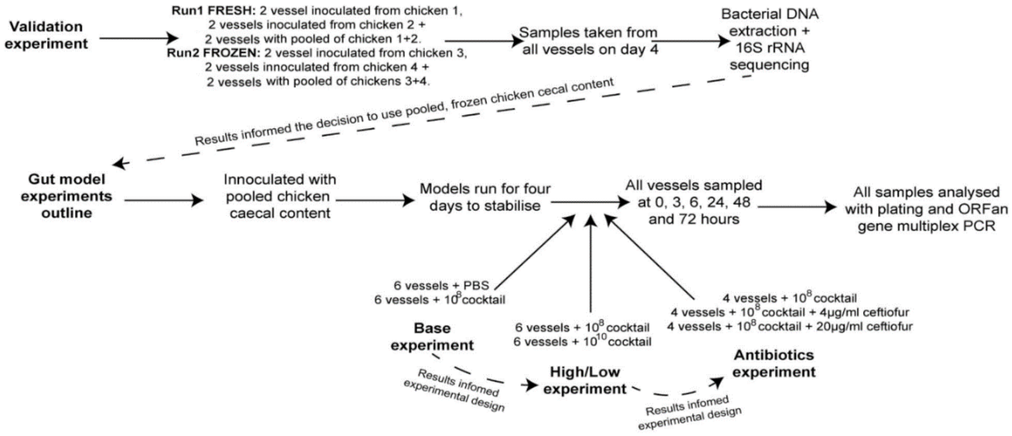

Supplementary item 8: Schematic representation of chicken caecal model experiments described in the paper.

Supplementary item 10: Volumes (µl) of each *E. coli* isolate used to make the 10^{10} *E. coli* cocktail. *E. coli* isolates were grown to approximately mid exponential phase and the volumes stated below mixed to create equal numbers of each isolate (5.88 x 10^8 cfu/ml) in the inoculum as described (32).

Isolate ID	Volume (µl)
Chicken1	102.9
Human1	410.4
Human2	379.5
Cattle1	275.7
Cattle2	430.4
Cattle3	98.3
Cattle4	284.6
Cattle5	100.6
Cattle7	426.8
Pig1	156.9
Pig2	735.3
Pig3	49.9
Pig4	1357.5
Pig5	296.6
Generalist4	181.0
Generalist3	732.3
Generalist1	60.4

Supplementary item 11: Primers used in four separate host-specific multiplex PCR assays to detect the presence of the 17 isolates of the *E. coli* cocktail. C/H, chicken and human associated assay; CA, cattle associated assay; PI, pig associated assay; GE, generalist assay.

Isolate ID	Multiplex PCR assay	Forward primers	Reverse primers	Product size (bp)
Chicken1	C/H	GGAAACGGATTACTTCTACG	CTGATAGAGATTCAGTCCC	793
Human1	C/H	CCTACGCCACTAAACTACTG	CTATCATCACTGGAAATCCTG	560
Human2	C/H	GAATTACCGTCGTAGAGCAG	GGTATGGACTCAATGACAC	970
Cattle1	CA	CTGCTGTTATTAATCGGCTTGG	CTCTCAAGCGTGCTTTCTATC	886
Cattle2	CA	GTGGTGTAGAATTTATCGCATCC	AGCCATTCGATGAAACCAAG	665
Cattle3	CA	GGGTCGATAACTTAGCCAAG	CTTGTTTGCAGAATGCTGCG	465
Cattle4	CA	GAATGGCAGGGCATACAAG	CAGACCTATGAACCTCTCCC	522
Cattle5	CA	CAGGAATGACAAACCTCTCG	CCGAACCATGCATTTGTCTG	1097
Cattle7	CA	CTGCGGAAGAGTGTAAGTTC	CAGCGTCATCACTAAGCATT	162
Pig1	PI	CGGAACTCCAGCGAAAG	CTCAGGTGCTTACTACGTTC	178
Pig2	PI	CCAGATAGAGTCGTTTCTGC	GCAGCATTAACAGTAGGTCC	434
Pig3	PI	GGCGATACGATTTTAACACCA	GCAACGGTCTAACATTCGCTG	347
Pig4	PI	GATATAGCAAAAGCCGTTTCCTG	GCCTAGCAATAAATAACCGGTC	564
Pig5	PI	GCTCGACATATTCCGAACAG	CACAGTTCTGGTGCAATAGAG	267
Generalist4	GE	CAGCAACGGATTGATACCTC	GCGAAGTTCTTCAATCTCC	697
Generalist3	GE	CAGTTATGCTGGGCTAATTG	TGCGTAATTTGCATGATATGG	485
Generalist1	GE	GGCCATTGATAGCAGCATTG	CCGAATAATAAATCGCC	371

Supplementary item 12: Table relating the labels used for the cocktail isolates in this paper to the bioproject, ID and SRA accession submitted to NCBI SRA.

Isolate ID from the paper	Isolate ID in NCBI SRA	NCBI Bioproject	SRA accession
Chicken1	21225_2#112	PRJA739205	SRR14866877
Human1	SAP1847	PRJA739205	SRR14867239
Human2	SAP1710	PRJA739205	SRR14867246
Cattle1	IMT38565	PRJNA926526	SAMN46777735
Cattle2	R45	PRJNA926526	SAMN46777736
Cattle3	IMT13936	PRJNA926526	SAMN46777737
Cattle4	IMT34414	PRJNA926526	SAMN46777738
Cattle5	IMT10909	PRJNA926526	SAMN46777739
Cattle7	9475_4#43	PRJNA926526	SAMN46777740
Pig1	IMT39234	PRJNA926526	SAMN46777741
Pig2	IMT28138	PRJNA926526	SAMN46777742
Pig3	39533	PRJNA926526	SAMN46777743
Pig4	IMT38723	PRJNA926526	SAMN46777744
Pig5	IMT38701	PRJNA926526	SAMN46777745
Generalist4	ZTA1601993EC	PRJA739205	SRR14866797
Generalist3	09-05726	PRJNA926526	SAMN46777746
Generalist1	21225_2#178	PRJA739205	SRR14866871
